# Geographic contrasts between pre‐ and postzygotic barriers are consistent with reinforcement in *Heliconius* butterflies

**DOI:** 10.1111/evo.13804

**Published:** 2019-09-11

**Authors:** Neil Rosser, Lucie M. Queste, Bruna Cama, Nathaniel B. Edelman, Florian Mann, Ronald Mori Pezo, Jake Morris, Carolina Segami, Patricia Velado, Stefan Schulz, James L. B. Mallet, Kanchon K. Dasmahapatra

**Affiliations:** ^1^ Department of Biology University of York Wentworth Way Heslington YO10 5DD United Kingdom; ^2^ Department of Organismic and Evolutionary Biology Harvard University Cambridge Massachusetts 02138; ^3^ Institut für Organische Chemie Technische Universität Braunschweig Hagenring 30 38106 Braunschweig Germany; ^4^ URKU Estudios Amazónicos Jr. Saposoa 181 Tarapoto, San Martín Perú; ^5^ Department of Ecology and Genetics Uppsala University Norbyvägen 18d 75236 Uppsala Sweden; ^6^ Department for Quality Assurance Analytics Bavarian State Research Center for Agriculture Lange Point 6 85354 Freising Germany

**Keywords:** Butterflies, gene flow, hybrid sterility, prezygotic isolation, speciation

## Abstract

Identifying the traits causing reproductive isolation and the order in which they evolve is fundamental to understanding speciation. Here, we quantify prezygotic and intrinsic postzygotic isolation among allopatric, parapatric, and sympatric populations of the butterflies *Heliconius elevatus* and *Heliconius pardalinus*. Sympatric populations from the Amazon (*H. elevatus* and *H. p. butleri*) exhibit strong prezygotic isolation and rarely mate in captivity; however, hybrids are fertile. Allopatric populations from the Amazon (*H. p. butleri*) and Andes (*H. p. sergestus*) mate freely when brought together in captivity, but the female F1 hybrids are sterile. Parapatric populations (*H. elevatus* and *H. p. sergestus*) exhibit both assortative mating and sterility of female F1s. Assortative mating in sympatric populations is consistent with reinforcement in the face of gene flow, where the driving force, selection against hybrids, is due to disruption of mimicry and other ecological traits rather than hybrid sterility. In contrast, the lack of assortative mating and hybrid sterility observed in allopatric populations suggests that geographic isolation enables the evolution of intrinsic postzygotic reproductive isolation. Our results show how the types of reproductive barriers that evolve between species may depend on geography.

Under a biological species concept, understanding speciation requires identifying the reproductive barriers between taxa and the order that they evolve (Coyne and Orr 2004; Butlin et al. 2012). However, which kinds of barriers evolve first may depend on geography (Coyne and Orr 1997). In the absence of gene flow, no forces inhibit speciation and populations can diverge through any combination of deterministic or stochastic processes, such as selection or drift (Turelli et al. 2001). Allopatric populations may therefore exhibit various combinations of prezygotic and postzygotic barriers, and postzygotic isolation can be either extrinsic or intrinsic. In general, the conditions for speciation are thought to become more restrictive as gene flow increases (Nosil 2007; Kisel and Barraclough 2010). For example, an important class of intrinsic postzygotic barriers among species are deleterious epistatic interactions between two or more divergent loci, known as Dobzhansky–Muller incompatibilities (DMIs; Orr and Turelli 2001). DMIs have often been viewed as unlikely to arise in the face of gene flow, because hybridization among diverging lineages produces double heterozygous genotypes with reduced fitness (Coyne and Orr 2004).

However, the constraining effects of gene flow can be reduced or even eliminated by the genetic architecture of the traits driving speciation (Maynard Smith 1966; Felsenstein 1981; Gavrilets 2004). For instance, DMIs may evolve as pleiotropic by‐products of divergent selection, if it is strong enough to outweigh the production of hybrids with low fitness (Bank et al. 2012). When matings among populations involve a cost, gene flow may even promote the evolution of reproductive isolation, because selection directly favors increased mate discrimination (Dobzhansky 1940; Servedio and Noor 2003). This process, known as reinforcement, may occur during sympatric speciation, or following secondary contact between populations derived in allopatry.

Empirical tests of these theoretical predictions require characterizing the components of reproductive isolation between closely related taxa with different levels of gene flow (Coyne and Orr 1997; Funk 1998; Funk et al. 2006). For example, under reinforcement it is expected that sympatric populations should exhibit stronger sexual isolation than allopatric populations. The most extensive comparative data in this respect are from *Drosophila*, where prezygotic sexual isolation accumulates more rapidly between sympatric than between allopatric pairs of taxa (Coyne and Orr 1997). This pattern is likely due to reinforcement (Yukilevich 2012), but whether it is evolving in response to intrinsic or extrinsic postzygotic isolation remains unclear (Turelli et al. 2014). Here, we characterize the specific traits contributing to reproductive isolation in allopatric, parapatric and sympatric populations of mimetic *Heliconius* butterflies.

*Heliconius* (Nymphalidae) comprises an adaptive radiation of ∼48 known species and 300+ subspecies with relatively well understood ecology, and provides excellent opportunities to study reproductive isolation among diverging populations in different geographical contexts (Jiggins 2017; Mérot et al. 2017). Previous studies of *Heliconius* close to the species boundary have typically found evidence of prezygotic isolation and/or extrinsic postzygotic isolation (McMillan et al. 1997; Chamberlain et al. 2009; Merrill et al. 2011a). For example, shifts in mimetic pattern are often thought to initiate speciation (Bates 1862), because interspecific hybrids displaying intermediate color patterns are selected against by predators (Merrill et al. 2012). Furthermore, because color pattern is itself used as a mating cue (Jiggins et al. 2001b), *Heliconius* provides prime examples of speciation facilitated by pleiotropy among traits under divergent selection and those involved in mate choice (Servedio et al. 2011). Nonetheless, the existence of closely related, sympatric taxa that do not differ in mimetic pattern suggests that mating cues other than color pattern are also important (Giraldo et al. 2008). For example, recent studies have demonstrated a role for pheromones in mediating mate choice (Mérot et al. 2015; Darragh et al. 2017). Divergent host plant and habitat use have also been proposed as sources of reproductive isolation (Estrada and Jiggins 2002; Rosser et al. 2019), and adaptations to environmental gradients have been linked to speciation (Jiggins et al. 1996; Mérot et al. 2013).

Gene flow is thought to play an important role in *Heliconius* evolution and may have allowed the adaptive transfer of mimetic color pattern alleles among species, possibly even leading to speciation (*Heliconius* Genome Consortium 2012; Pardo‐Díaz et al. 2012; Zhang et al. 2016 but see Brower 2018). One example of this is *Heliconius elevatus* and *Heliconius pardalinus*. *Heliconius elevatus* is characterized by a red, black, and yellow “rayed” pattern, which it shares with *Heliconius erato*, *Heliconius melpomene*, and many other Heliconiini. In contrast, *H. pardalinus* exhibits a mottled brown, black, and orange “tiger” pattern that mimics similarly patterned Ithomiini, as well as other Heliconiini. Introgression of color pattern alleles between *H. melpomene* and the common ancestor of *H. pardalinus* and *H. elevatus* at two key loci appears to have triggered the switch to a rayed pattern (*Heliconius* Genome Consortium 2012; Wallbank et al. 2016). Contemporary gene flow between *H. elevatus* and *H. pardalinus* has yet to be estimated, although wild‐caught putative hybrids (Brower 2018) and the fertility of lab‐reared hybrids (see below) suggest that it does occur.

In the present paper, we characterize an extensive set of phenotypic traits potentially involved in prezygotic and intrinsic postzygotic isolation among populations of *H. elevatus* and *H. pardalinus* in northern Peru. Broad‐scale distribution maps show that *H. elevatus* overlaps with the subspecies *H. p. butleri* in the Amazonian lowlands (Rosser et al. 2012; Fig. 1). A different subspecies, *H. p. sergestus*, inhabits the upper Huallaga/Mayo valleys in the adjacent Andes, where *H. elevatus* is absent. The two *H. pardalinus* subspecies have diverged in their tiger color pattern to mimic different co‐occurring ithomiine butterflies (Fig. 2). Phylogenetic analysis of these populations using genome‐wide single nucleotide polymorphisms shows *H. p. sergestus* to be sister to a clade containing *H. elevatus* and *H. p. butleri* + *H. p. dilatus* (the latter two are closely related adjacent populations from the Peruvian Amazon, and are hereafter referred to collectively as *H. p. butleri*), thus rendering *H. pardalinus* as a whole paraphyletic (*Heliconius* Genome Consortium 2012 and Fig. 2A). In light of this paraphyly and geographic distribution of the taxa, in the present paper we address the following questions: (1) Which specific traits contribute to reproductive isolation? (2) Do the geographic patterns of prezygotic and postzygotic isolation suggest speciation with gene flow? (3) Where are the species boundaries in these taxa?

**Figure 1:**
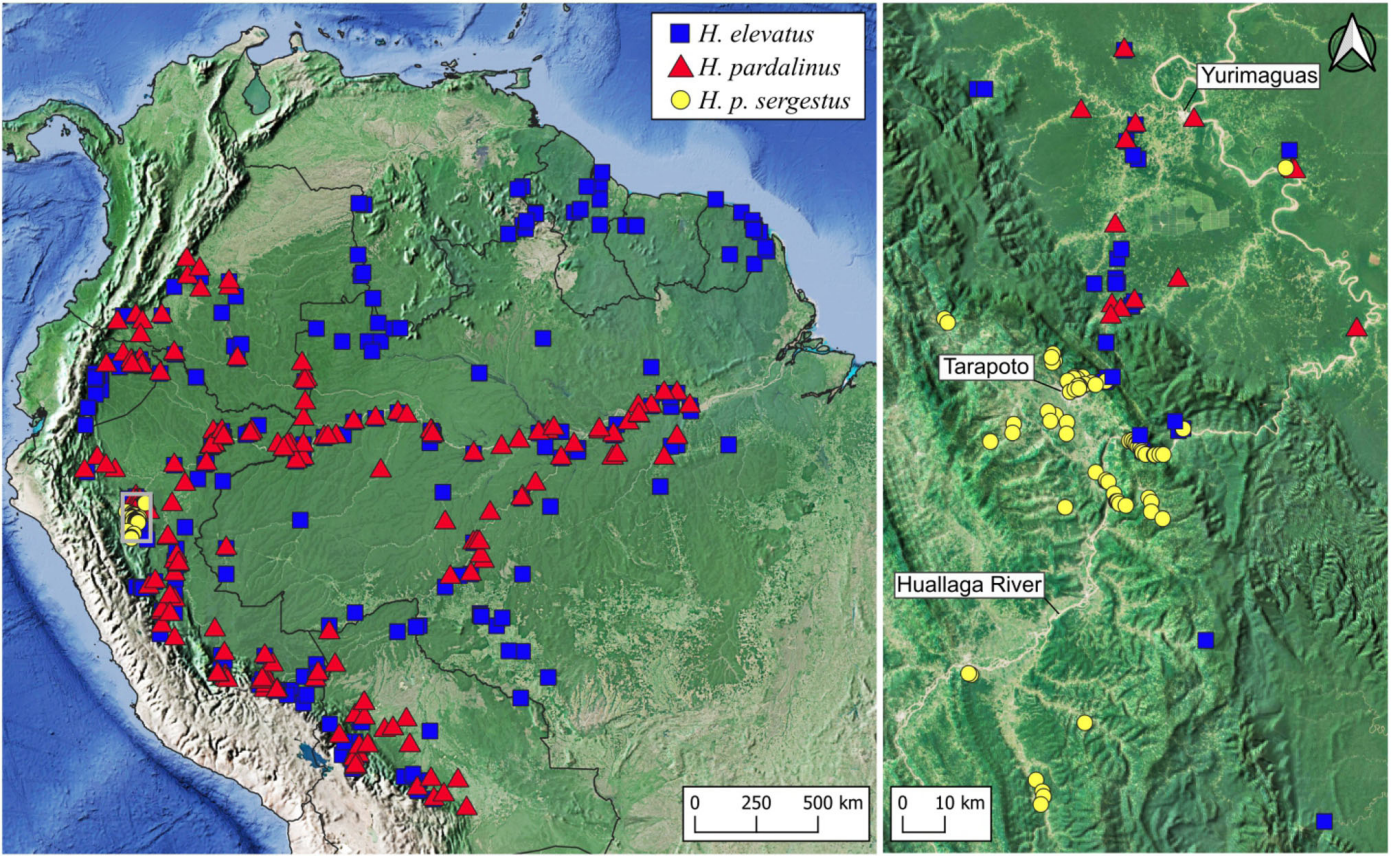
Left panel: The geographic distributions of *H. elevatus* and *H. pardalinus* (all Amazonian subspecies) at a continental scale, with the range of subspecies *H. p. sergestus* shown in yellow. Right panel: Local map showing the fine scale distributions in northern Peru, centered on the range of *H. p. sergestus*. In this map, the red triangles correspond to the subspecies *H. p. butleri*, which intergrades into other, similarly patterned subspecies in lowland Amazonia. Data are taken from Rosser et al. (2012) and supplemented with newer field collections made by the authors (see Methods and Results sections).

**Figure 2:**
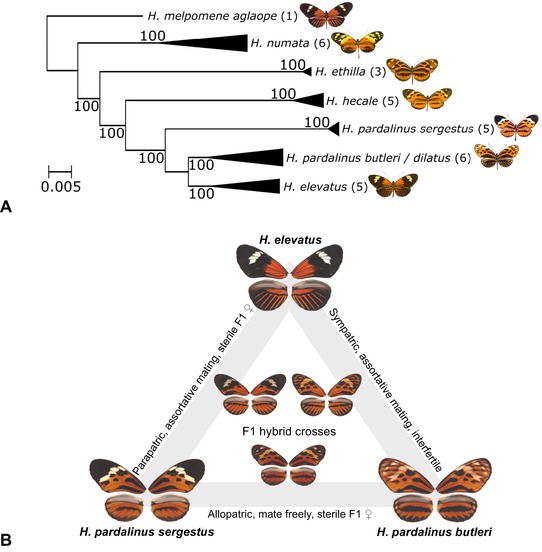
(A) Maximum‐likelihood phylogeny for the Peruvian silvaniform taxa, with *H. melpomene aglaope* as the outgroup, based on restriction site associated DNA (RAD) sequences (Supplementary Information S1). The scale bar refers to the number of substitutions per site, and node values are bootstrap support. Figures in brackets indicate the number of samples. *Heliconius p. butleri* clustered with the subspecies *H. pardalinus dilatus* from central Peru; the two are very similarly patterned and gradually intergrade. (B) Color patterns of the three parental taxa and their F1 hybrids, together with a summary of their relative geographic distributions and reproductive compatibility.

## Methods

Live butterflies collected in the Peruvian departments of San Martín, Loreto, and Ucayali were used to establish butterfly stocks in outdoor insectaries in Tarapoto, Peru, and heated indoor insectaries in York, UK. Adult butterflies were fed sugar/pollen solution and provided with additional pollen sources such as *Lantana camara* (Verbenaceae), *Gurania* sp. (Cucurbitaceae), and *Polianthes tuberosa* (Asparagaceae). Larvae were fed primarily using *Passiflora caerulea*, *P. edulis*, *P. riparia*, and *P. serrato‐digitata* (Passifloraceae). Experiments involving *H. elevatus*, *H. p. butleri*, and *H. p. sergestus* used a mixture of wild and captive‐bred butterflies. Crosses between the taxa were produced in the insectaries, through either natural matings or handpairing (Clarke and Sheppard 1956, and see Supplementary Information S2). Statistical analyses were carried out in R (R Core Team 2018) using base functions, unless otherwise stated.

### PREZYGOTIC ISOLATION: GEOGRAPHY, HABITAT, AND CLIMATE

Geographic barriers or divergent adaptations that prevent populations from encountering one another can be important sources of reproductive isolation (Kirkpatrick and Ravigné 2002; Coyne and Orr 2004; Sobel et al. 2010). To determine the local distributions and habitats of *H. elevatus*, *H. p. butleri*, and *H. p. sergestus* near Tarapoto, we made extensive collections during 2009–2016. To quantify the climatic niche of each taxon, we obtained 30 arcsec (1 km^2^ resolution) gridded climate data (WorldClim version 1; Hijmans et al. 2005). We then used ArcGIS 10 to extract mean annual temperature values and annual precipitation values for each collection locality.

### PREZYGOTIC ISOLATION: FEMALE HOST PLANT PREFERENCE

Host plant shifts have long been recognized as holding the potential to create reproductive isolation in phytophagous insects, especially when mating occurs on or near the host plant (Ehrlich and Raven 1964; Bush 1969). To investigate whether *H. elevatus*, *H. p. butleri*, and *H. p. sergestus* differ in host plant use, we recorded field observations in Peru, Bolivia, Brazil, Suriname, and French Guiana, and supplemented these with records from the literature. However, such data are hard to obtain, and furthermore, may simply reflect which host plants are available to local populations of butterflies, rather than divergent adaptations between them. We therefore conducted laboratory experiments in Peru to test for differences in host plant preference. Reared and wild caught females of a single taxon were released into a large cage (2.5 m [W] × 5 m [L] × 2 m [H]) containing 21 species of *Passiflora* (Table S1) commonly found near Tarapoto and representing potential host plants. Groups of 3–33 females from a single taxon were taken at random from stocks and left to oviposit in this cage for up to 7 days. At the end of each day, the number of eggs laid on each plant species was recorded, and the eggs removed. To reduce the effects of individual variation in female preference and host plant quality, each butterfly taxon was tested repeatedly over several months. To measure similarity in host plant use, we calculated pairwise values of Pianka's (1973) niche overlap index for the three taxa, using the number of eggs laid across the 21 host plants. The index varies from zero (when no resources are shared) to one (when resource use is identical).

We also conducted a second experiment to test for differences in host plant preference while directly controlling for variation in individual preference and host plant size/quality. Single females were introduced into a cage measuring 1 m (W) × 2 m (L) × 1.7 m (H), with four approximately equally sized shoots of potential host plants (*P. edulis*, *P. laurifolia*, *P. riparia*, and *P. serrato‐digitata*) placed in each corner of the cage. At the end of each day, the number of eggs laid on each plant species was recorded and the eggs removed. For each pairwise comparison of taxa, we used Generalized Linear Mixed Effect Models (GLMM) with negative binomial errors to test for differences in the number of eggs laid on each plant, using the R package lme4 (Bates et al. 2015). Host plant species and butterfly taxon were specified as fixed effects, and individual as a random factor. Two nested models were fit for each of the three pairwise comparisons, one including the interaction among fixed effects (i.e., evidence of a difference in species preference) and one without. Models were tested against one another using ANOVA, and the Akaike information criterion (AIC) was used for model selection.

### PREZYGOTIC ISOLATION: MALE COLOR PATTERN PREFERENCE

An important mating cue for male *Heliconius* is female wing color pattern (Jiggins et al. 2001b; Merrill et al. 2011a). To test whether *H. elevatus*, *H. p. butleri*, and *H. p. sergestus* males exhibit a preference for their own color pattern phenotype, we measured courtship effort by males when given a choice of female wings, one bearing their own phenotype and the other bearing an alternative phenotype. We then used Generalized Linear Models (GLMs) with binomial errors to estimate the predicted probability of a male courting its own phenotype or the alternative, with a categorical predictor indicating the six pairwise comparisons.

Experiments were conducted in Peru and the UK using the experimental setup shown in Figure S1. In the experiments in the UK, male preference data were collected only for *H. elevatus* and *H. p. butleri*. Groups of five males (either *H. elevatus* or *H. p. butleri*) were presented with a pair of model wings (one *H. elevatus* and one *H. p. butleri*), and trials lasted for 25 min. The number of approaches (clear, directed flights to within 10 cm of a model), hovers (sustained flight 5–15 cm over a model), and alightings (landing on or next to a model) by the males directed toward each of the model wings was recorded (Klein and de Araújo 2010). After a courtship event, the male was caught and its identity recorded. In Peru, we used pairs of males (representing two of the three taxa) to avoid having to catch individuals after each courtship. Males were presented with two female wing models exhibiting the corresponding color patterns and placed in the experimental cage 1 day before testing to allow acclimatization. Courtship trials lasted 15–30 min.

### PREZYGOTIC ISOLATION: MALE SEX PHEROMONES

Sex pheromones are a potentially important source of sexual/behavioral prezygotic reproductive isolation because they can be used as a cue for mate choice (Smadja and Butlin 2009). In butterflies, male sex pheromones are mostly emitted from specialized scales on the wings (Rutowski 1980), known as androconia. In male *Heliconius*, the androconia are most strongly concentrated on the anterior margin of the dorsal hind wing (Emsley 1965), and the volatiles they produce are involved in female mate choice (Darragh et al. 2017). We therefore tested the male androconia of *H. elevatus*, *H. p. butleri*, and *H. p. sergestus* for differences in the putative pheromone compounds. Dichloromethane extracts from the androconial region were taken from males of 10 *H. elevatus*, 13 *H. p. butleri*, and 5 *H. p. sergestus* (Fig. S2). Control samples of the non‐androconial region on the posterior margin of the hind wing were also taken from five of the males of each taxon. In addition, control samples of the anterior margin of the hind wing were taken from two *H. elevatus* and two *H. p. butleri* females (no *H. p. sergestus* females were sampled). All butterflies were ∼21 days old.

Samples were analyzed by gas chromatography–mass spectrometry (GC–MS). Tridecyl acetate was used as internal standard so the amount (nmoles) of each compound in each sample could be calculated. Compounds produced by butterflies were identified through comparison of mass spectra and gas chromatographic retention indices with synthetic samples and mass spectrometric databases (see Mann et al. 2017 for full details). One *H. elevatus* non‐androconial control showing signs of contamination was discarded. Compounds were classed as putative male sex pheromone components if they were present in greater amounts in the male androconial region than either male nonandroconial controls (significance determined using Wilcoxon signed rank tests), female controls (significance determined using Mann–Whitney *U*‐tests), or both. This putative male sex pheromone dataset was reduced to two dimensions by nonmetric multidimensional scaling (NMDS) ordination with a Bray–Curtis similarity matrix, using the vegan R package (Oksanen et al. 2017). For this, we used the proportion of compounds found for each individual. Finally, we carried out an analysis of similarities with the nonparametric ANOSIM to test whether the three taxa exhibited different pheromone profiles.

### PREZYGOTIC ISOLATION: ASSORTATIVE MATING

To test for the presence of prezygotic barriers that prevent *H. elevatus*, *H. p. butleri*, and *H. p. sergestus* from mating in the event that they encounter one another, we presented single virgin females to groups of males of the three taxa. The experiments are not intended as an accurate simulation of the butterflies mating behavior in the wild (in fact, *H. p. butleri* and *H. p. sergestus* very rarely encounter each other, see below). Moreover, the strength of assortative mating is the product of male and/or female choice, and represents the sum effect of multiple potential barriers (e.g., pheromones and color pattern preference).

Males comprised groups of three (one of each taxon) or 15 (five of each taxon) individuals, and were at least 1‐week old to ensure sexual maturity. Experiments were monitored hourly to catch mating pairs and lasted up to 5 days, although most matings occurred in the first few hours. Females were also checked regularly for the presence of spermatophores in case a mating had occurred but not observed. In the event of an observed mating, the mating pair was replaced and not reused. In the event a mating occurred but was not observed, all the butterflies were replaced. The log likelihood of a female of a given taxon mating with an *H. elevatus*, *H. p. butleri*, or *H. p. sergestus* male was calculated as

$$\;\log_{e}\left(L\left(P_{i}|n,y_{i}\right)\right)=\sum_{i=1}^{k}y_{i}\log_{e}\left(P_{i}\right)\;,$$

where *P_i_* is the probability of a type *i* mating, *y_i_* is the number of type *i* matings, *n* is the number of total matings, and *k* is the number of different mating types (3). Support limits for *P_i_* were obtained by finding all sets of parameter values with log*_e_* likelihoods within two units of the maximum likelihood estimate (Edwards 1972). To test for an effect of the number of males in the experiment, we used a likelihood ratio test to compare the mating probabilities estimated separately for experiments with three and 15 males (four parameters) with those estimated combining the experiments (two parameters).

### PREZYGOTIC ISOLATION: MALE COURTSHIP BEHAVIOURS

To test for taxon‐specific differences in male preference alone, we counted stereotyped courtship behaviors (Klein and de Araújo 2010) exhibited by males toward females during the assortative mating trials involving 15 males (five of each taxon) and a single virgin female. For 15 min every hour between 10 a.m. and 3 p.m., we recorded the numbers of approaches, hovers, and alightings of males toward the female. To obtain estimates of the number of courtship events per female for the three behaviors, for each of the three taxa we fitted GLMMs with negative binomial errors to account for overdispersion and with number of courtship events by males as the dependent variable (for each type of courtship, giving nine models in total) using the R package lme4 (Bates et al. 2015). Male taxon was included as the independent variable and individual female as a random effect.

### POSTZYGOTIC ISOLATION: EGG HATCH RATE AND PUPAL SURVIVORSHIP

An important source of intrinsic postzygotic isolation resulting from genetic incompatibilities among taxa is sterility and reduced viability of hybrids. To test for this effect, we measured egg hatch rate and pupal survivorship of crosses within and among the three taxa, including backcrosses and F1 × F1 crosses (Tables 4 and 5). Experiments were conducted in our Peruvian insectaries. For egg hatch rate, eggs were initially collected at the end of each day, and those from a single female housed together in plastic containers. However, egg parasitism by *Ooencyrtus* sp. near *marcelloi* (det. John Noyes, May 2015) and possibly cannibalism resulted in lower measured hatch rates for within species crosses than found in previously published studies (McMillan et al. 1997; Jiggins et al. 2001a; Naisbit et al. 2002). Subsequently, egg collection was carried out every 2 hours between 9 a.m. and 5 p.m., and eggs housed in individual plastic containers. If an egg did not hatch after 7 days from the date of collection, it was inspected under a microscope for the presence of parasitoids. If an egg parasitoid was found, the egg was excluded from hatch rate calculation.

To test for variation in hatch rate, logistic regression was used to model the proportion of eggs hatching. We began by testing for an association between hatch rate and a binary predictor indicating whether eggs were collected before or after the change in protocol. We then added cross type as a predictive factor to this model and tested whether its inclusion significantly improved the fit, using likelihood ratio tests. Differences in survival between replicate broods due to unaccounted genetic or environmental variation led to higher variance than can be explained by a binomial distribution. Therefore, the variance was specified as V(μ)=Φμ(1−μ), where *μ* is the mean and Φ the dispersion parameter.

We also tested for hybrid inviability in pupae by recording the survival of pupae of seven cross types (Table 5). Survival was recorded as either (1) successful emergence of a butterfly, (2) failed emergence from the pupa, (3) nothing emerged from the pupa, or (4) prepupa failed to form a pupa. Information on brood identity was not available, and therefore we were not able to account for between‐brood variance as was done for egg hatch rate.

### QUANTIFYING ISOLATION

We followed the method presented by Sobel and Chen (2014) to quantify the level of reproductive isolation (*Ri*) caused by each trait, using the formula:

Ri=1−2x,

where *x* is the probability of gene flow, which can be calculated for each trait. *Ri* is a relative measure where *Ri* = 0 implies random mating, *Ri* = 1 represents complete assortative mating, and *Ri* = –1 complete disassortative mating. The calculation of *x* depends on the trait being considered and is detailed in Supplementary Information S6.

## Results

### PREZYGOTIC ISOLATION: GEOGRAPHY, HABITAT AND CLIMATE

**Figure 3:**
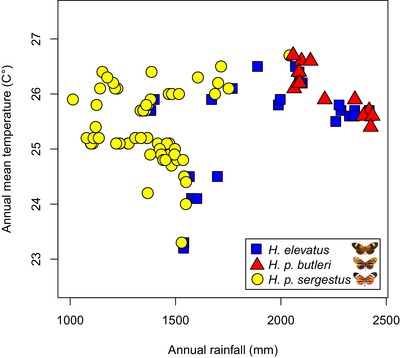
Observed climatic niches of *H. elevatus* (blue squares), *H. p. butleri* (red triangles), and *H. p. sergestus* (yellow circles) along rainfall (mm) and temperature (°C) gradients.

Geographic data show the lowland subspecies of *H. pardalinus* are sympatric with *H. elevatus* at a broad scale across Amazonia (Fig. 1). However, at a fine scale our field collections suggest that the two exhibit habitat segregation, with *H. elevatus* typically encountered in tall, well‐drained, ridge‐top forest, and *H. pardalinus* more commonly found in swampy, low‐lying areas with scrubby vegetation (see also Brown 1976). Nonetheless, we have observed the two flying together at three sites (Muniches and Micaela Bastidas, both near Yurimaguas, in Peru and Careiro Castanho, south of Manaus in Brazil). The ranges of *H. p. butleri* and *H. p. sergestus* are separated by the Cordillera Escalera, which lies between the upper Huallaga/Mayo valley and Amazonian lowlands (Fig. 1). The two occupy very different habitat types, with *H. p. sergestus* primarily occurring in tropical dry forest created by the rain shadow of the cordillera. *Heliconius p. sergestus* is notable for exhibiting extreme temporal variations in abundance. In 2016, the *H. p. sergestus* population increased to such a degree that specimens were collected in the Amazon lowlands, flying together with *H. p. butleri* (Fig. 1). Even including this extreme event, the known distribution of *H. p. sergestus* is highly restricted, with a maximum linear extent of 160 km. Unlike *H. p. butleri*, *H. elevatus* inhabits the Cordillera Escalera up to 1000 meters and reaches the ecotone to the dry forests where *H. p. sergestus* occurs. The climatic niches of the three taxa are shown in Figure 3 and reflect these geographic distributions; *H. elevatus* and *H. p. butleri* overlap in their climatic niches, but *H. elevatus* also inhabits cooler and drier environments than *H. p. butleri*. *Heliconius p. sergestus* and *H. p. butleri* exhibit more marked divergence in their climatic envelopes, with segregation along the rainfall gradient.

### PREZYGOTIC ISOLATION: FEMALE HOST PLANT PREFERENCE

Near Tarapoto, we have recorded *H. elevatus* ovipositing on *P. laurifolia*, *P. coccinea*, and *P. vitifolia*. However, its most important host plant is a large, canopy growing species in the Laurifoliae group, from here on referred to as *P. *(Laurifoliae) sp. We have also recorded *H. p. sergestus* and *H. p. butleri* ovipositing on *P. laurifolia* (and closely related variants of it). Elsewhere in the Amazon basin, *H. elevatus* has been recorded ovipositing on *P. laurifolia* and *P. longiracemosa*, and populations of *H. pardalinus* have been recorded ovipositing on *P. coccinea*, *P. spinosa*, and *P. nitida* (NR and JLBM, pers. obs.; Benson et al. 1975). Detailed notes summarizing what is known about the wild host plant use of these taxa are given in Supplementary Information S4.

**Figure 4:**
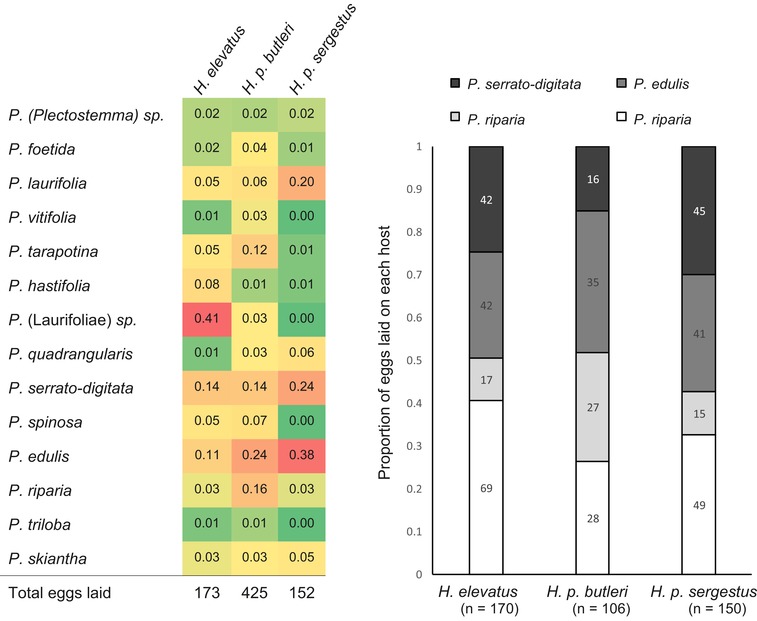
Host plant preference of the three taxa. (A) Preference measured as the proportion of eggs laid by multiple females on 21 species of *Passiflora* (Table S1) commonly occurring near Tarapoto and representing potential host plants. Seven plant species were not oviposited on and are not shown. (B) Preference measured as the proportion of eggs laid on size/quality matched shoots of four *Passiflora* species. In brackets is the total number of eggs laid by each taxon. 12 *H. elevatus*, 12 *H. p. butleri*, and 10 *H. p. sergestus* females were tested. Numbers within each column show the number of eggs laid on each host plant.

In the first host plant experiment, 51 *H. p. butleri* females laid 425 eggs on 16 species of host plant; 37 *H. p. sergestus* females laid 162 eggs on 10 species of host plant; and 34 *H. elevatus* females laid 173 eggs on 14 species of host plant. The plant most frequently used by *H. p. butleri* was *P. edulis*, on which it laid 24% of its eggs. *Passiflora edulis* was also the plant most frequently used by *H. p. sergestus* (38% of eggs laid). Consistent with our observations in the wild, the plant most frequently used by *H. elevatus* was *P. *(Laurifoliae) sp. (41% of eggs laid). The full results of this experiment are shown in Figure 4 and Table S1. Pianka's niche overlap coefficient showed high similarity in host plant use between *H. p. butleri* and *H. p. sergestus* (*O* = 0.81), whereas host plant overlap was less between *H. elevatus* and *H. p. butleri* (*O* = 0.47) and least between *H. elevatus* and *H. p. sergestus* (*O* = 0.38).

In the second host plant experiment, we tested the preferences of females across four host plant species using a mixed effect model to account for variation in individual preference, and using shoots of equal size/quality. A total of 170, 106, and 150 eggs were laid by 14 *H. elevatus*, 13 *H. p. butleri*, and 10 *H. p. sergestus* females, respectively. *Heliconius elevatus* laid 41% of its eggs on a single host (*P. laurifolia*), and *H. p. butleri *and *H. p. sergestus *both laid 33% of their eggs on their preferred hosts, *P. edulis* and *P. serrato‐digitata*, respectively (Fig. 4). We found a significant interaction between butterfly taxon and host plant species when comparing *H. elevatus* and *H. p. butleri*, indicating different host plant preferences (*P* = 0.02; ∆AIC = 3.7), and a marginally nonsignificant interaction when comparing *H. p. sergestus* and *H. p. butleri* (*P* = 0.06; ∆AIC = 1.4). We found no significant interaction when comparing *H. elevatus* and *H. p. sergestus* (*P* = 0.94; ∆AIC = 5.6).

### PREZYGOTIC ISOLATION: MALE COLOR PATTERN PREFERENCE

One hundred and sixty‐seven males were tested for color pattern preference. Data for all courtship events are given in Figure S3. Here, we restrict our results to hovers (591 events, performed by 119 individuals) as this behavior is the most unambiguous sign of courtship. For each taxon, the estimated probabilities and 95% confidence intervals of courting the conspecific model are given in Table 1. Initially, we included a binary predictor corresponding to whether data were collected in Peru or the UK; however, as no significant difference was found (*P* = 0.91), these datasets were combined. *Heliconius elevatus* showed a significant preference for its own phenotype when presented with models of itself and either *H. p. butleri* or *H. p. sergestus*. *Heliconius p. butleri* also showed a significant preference for its own phenotype when presented with models of itself and *H. p. sergestus*, but courted its own phenotype and the *H. elevatus* phenotype about equally. *Heliconius p. sergestus* showed no statistically significant preference for any color pattern phenotype.

**Table 1:** Male color pattern preference. Male butterflies were presented with conspecific and heterospecific models of female butterflies. The table shows the estimated probabilities (±95% confidence intervals) from GLMs of a male showing hovering courtship behavior toward its own color pattern relative to the other. Predicted probabilities significantly different to 0.5 (i.e., showing significant preference) are shown in bold; *n* is the number of hovers performed, and the number of individuals tested is shown in brackets

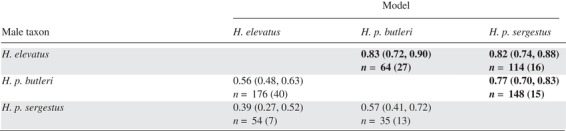

### PREZYGOTIC ISOLATION: MALE SEX PHEROMONES

**Figure 5:**
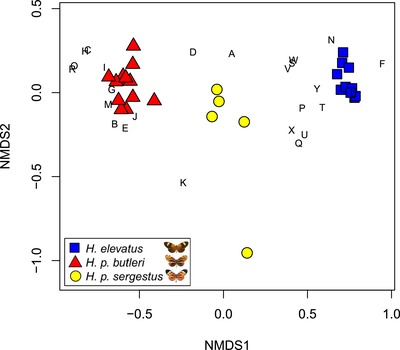
Taxa (represented by symbols) and compounds (represented by letters) along the first two dimensions of the NMDS ordination of 25 putative sex pheromone compounds found in hind‐wing androconia of males. Axes represent gradients of similarity among samples (similarity in compound composition) and among compounds (similarity in relative abundance across samples). A, homovanillyl alcohol; B, hexahydrofarnesylacetone; C, ?‐eicosene; D, ??‐heneicosadiene; E, (*Z*)‐9‐heneicosen; F, heneicosane; G, ?‐docosene; H, oleyl acetate; I, octadecyl acetate; J, phytol; K, (*Z*)‐9‐tricosene; L, tricosane; M, (*Z*)‐11‐eicosenyl acetate; N, tetracosane; O, (*Z*)‐11‐eicosenyl propionate; P, pentacosane; Q, 11‐methylpentacosane; R, (*Z*)‐13‐docosenyl acetate; S, hexacosane; T, 11‐methylhexacosane; U, heptacosane; V, 11‐methylheptacosane; W, octacosane; X, nonacosane; Y, octacosanal (? indicates unknown position of double bond).

GC–MS analysis detected 53 compounds from samples representing 28 male individuals of *H. elevatus*, *H. p. butleri*, and *H. p. sergestus* (10, 13, and 5, respectively) and two female controls of *H. elevatus* and *H. p. butleri* each. Thirteen compounds were excluded because they were likely contaminants or because they only appeared once in the dataset. Male androconia were found to contain more compounds, and in larger quantities, than both male hind wing and female controls (Table 2). Thirty‐three of the 40 retained compounds were present in significantly different amounts in at least one of the pairwise comparisons between taxa (Table S2; details of the species‐specific chemical blends are provided in the Supplementary Information S5). Finally, 25 of the compounds were found in significantly higher concentrations in the male androconia compared to either the male hind wing control or the female control (or both). When plotted, the results from the NMDS analysis of these 25 compounds show that the three taxa form nonoverlapping groups along NMDS axis 1 (Fig. 5); *H. elevatus* and *H. p. butleri* individuals cluster at opposite ends of this axis and *H. p. sergestus* individuals cluster in between. The chemical composition of the taxa was significantly different (ANOSIM *R* = 0.97, *P* = 0.001).

**Table 2:** Summary of putative pheromone compounds detected by GC–MS analysis of wing extracts. Values shown are the median amount (interquartile range) in nmol of total compounds found in extracts from male androconia, male hind wing controls, and female controls of all three taxa, and *n* is the average number of detectable compounds in each extract. Mann–Whitney *U*‐test results are presented for each male androconia/control comparison of total concentration of compounds; male androconia have significantly higher total concentrations. No female control samples were analyzed for *H. p. sergestus*

	Male androconia			Control regions		
Taxon	Total (nmol)	*n*		Total (nmol)	n	P‐value
*H. elevatus*	18.6 (15.6–22.4)	21.9	Male hind wing control	3.9 (1.4–1.8)	15.7	0.0002
			Female control	4.4 (4.1–4.7)	17	0.03
*H. p. butleri*	39.9 (37.3–49.0)	30.7	Male hind wing control	1.7 (1.4–1.8)	12.6	3 × 10^–5^
			Female control	1.5 (1.4–1.6)	9	0.02
*H. p. sergestus*	8.1 (7.9–10.2)	23.8	Male hind wing control	2.2 (2.1–2.5)	13.9	0.008
			Female control	–	–	–

### PREZYGOTIC ISOLATION: ASSORTATIVE MATING

We presented 161 virgin females to males of *H. elevatus*, *H. p. butleri*, and *H. p. sergestus*, resulting in 44 matings over 253 trials (Table 3). *Heliconius elevatus* females mated only with *H. elevatus* males (*n* = 13). *Heliconius p. butleri* females mated with both *H. p. butleri* (*n* = 13) and *H. p. sergestus* (*n* = 10) males, but not with *H. elevatus*. Similarly, *H. p. sergestus* females mated with both *H. p. butleri* (*n* = 5) and *H. p. sergestus* (*n* = 3), but not with *H. elevatus*. The maximum likelihood parameter estimates for mating probabilities with associated support limits are given in Table 3. Analyzing experiments using three males and 15 males separately did not significantly improve the likelihood estimates, and hence data from the two experiments were combined (likelihood ratio tests: for *H. elevatus* females *χ*² = 0, df = 2, *P* =  NS; for *H. p. butleri* females *χ*² = 0.44, df = 2, *P* = NS; for *H. p. sergestus* females *χ*² = 0, df = 2, *P* = NS).

**Table 3:** Results from assortative mating trials between the three taxa. Single virgin females were presented to equal numbers of *H. elevatus*, *H. p. butleri*, and *H. p. sergestus* males, and the number of matings (*n*) recorded. Numbers in brackets after the female taxa give the total number of virgin females tested (not all of which mated). The *P* column is the maximum likelihood estimate of the probability of mating with a male of each taxa, with support limits in brackets

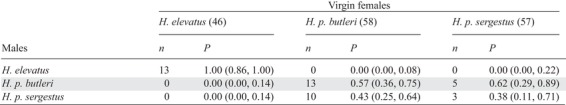

### PREZYGOTIC ISOLATION: INTRA‐ AND INTERTAXON COURTSHIP BEHAVIOURS

**Figure 6:**
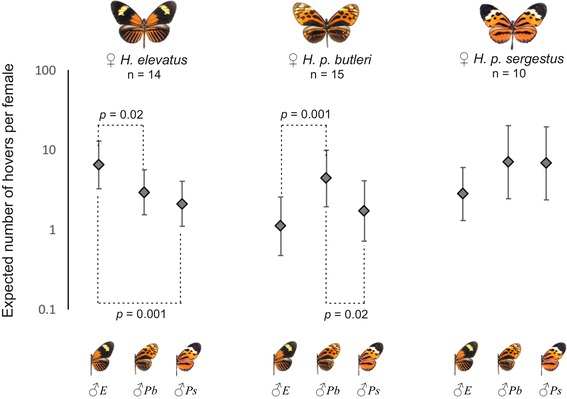
Assay of hovering courtship behavior within and between taxa. Single female virgins were presented to groups of 15 males (five of each taxon) and hover courtship toward the female was recorded. The expected number of hover courtship behaviors per trial by males toward the female taxa and the statistical significance of any differences were obtained from GLMM model outputs. Error bars are 95% Wald confidence intervals; *n* is the number of virgin females tested of each taxon; *E*, *H. elevatus*; *Pb*, *H. p. butleri*; *Ps*, *H. p. sergestus*. Results of the other courtship behaviors measured (approaches and alightings) are shown in Figure S4, and details of the significance values following Bonferroni correction are provided in Table S3.

During behavioral assays of male courtship, we recorded a total 388 approaches, 616 hovers, and 105 alightings. Data for all courtship events are given in Table S3 and Figure S4. Figure 6 shows the expected numbers of hovers per trial received by females of each taxon from males of the three taxa, as output from the GLMMs. *Heliconius elevatus* males hovered over *H. elevatus* females significantly more than *H. p. sergestus* and *H. p. butleri* males (although this was no longer significant for the latter after using the conservative Bonferroni correction method, see Table S3). *Heliconius p. butleri* males hovered over *H. p. butleri* females significantly more than *H. elevatus* and *H. p. sergestus* males. In contrast, *H. p. sergestus* males did not hover over *H. p. sergestus* females significantly more than the males of the other taxa.

### POSTZYGOTIC ISOLATION: EGG HATCH RATE AND PUPAL SURVIVORSHIP

Data on egg hatch rate were collected from 4,423 eggs from 110 broods (Table 4). We observed taxon‐specific differences in fecundity between the taxa, with female *H. elevatus* and *H. p. sergestus* laying fewer eggs per day on average than *H. p. butleri* (Supplementary Information S6). Female F1s (*n* = 25) produced by crossing *H. p. butleri* and *H. p. sergestus* in either direction laid no eggs, and dissection of female gonads confirmed them to be sterile, with ovaries lacking maturing eggs. F1 females produced by crossing either *H. p. sergestus* males with *H. elevatus* females (*n* = 3) or *H. elevatus* males with *H. p. sergestus* females (*n* = 2) were also sterile. Female F1s produced by crossing *H. p. butleri* and *H. elevatus* were found to be fully fertile in both directions, with intermediate fecundity and with no significant differences in egg hatch rate when compared with pure females. Table 4 shows the predicted hatch rates and confidence intervals for each possible cross without parasitism.

**Table 4:** Estimated egg hatch rates for crosses within and between taxa; E = H. elevatus, Pb = H. p. butleri, Ps = H. p. sergestus. F1 genotypes comprise the mother's identify followed by the father's, i.e. female “Pb × E” had a H. p. butleri mother and a H. elevatus father. Hatch rates (hatch) are estimated assuming no parasitism, and include 95% confidence intervals (in brackets). *n* = the number of broods per cross and mean brood size (in brackets). Fertile? ^*^ indicates that the crosses appear to be fertile on the basis of crosses made outside of controlled experiments.^†^ sterile crosses; female F1s with genotypes Ps × Pb, Pb × Ps, Ps × E and E × Ps were found to be sterile with undeveloped ovaries. ^**^ the number of females dissected to determine the status of ovary development

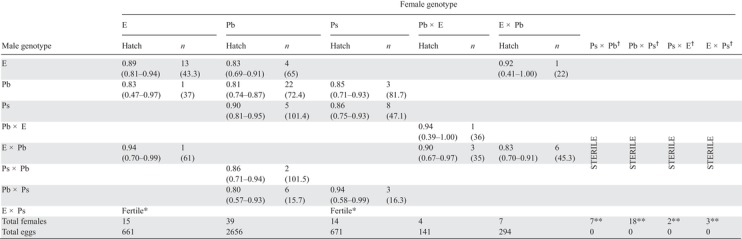

We also tested pupal survivorship of within taxon (pure) and between taxon (hybrid) crosses from a total of 844 pupae (Table 5). Although we found some evidence for variation in pupal survivorship across cross types (test for equality of proportions; *χ*² = 29.21, df = 6, *P* < 0.001), F1 and F2 individuals had equivalent or higher survivorship than pure crosses. This suggests there are no strong reductions in pupal survivorship attributable to hybrid incompatibilities. Therefore, aside from sterility in female F1s between *H. p. sergestus* and either *H. p. butleri* or *H. elevatus*, we found no evidence for sterility or reductions in viability of between‐taxon crosses. Additional observations on the life history and immature stages of *H. elevatus*, *H. p. butleri*, and *H. p. sergestus* are given in Supplementary Information S6.

**Table 5:** Survival of pupae from seven cross types. See Table 4 legend for the codes of each cross type; *n* is the number of pupae; $\hat{p}$ is the proportion of individuals, with 95% confidence intervals provided for the proportion of successful emergences

	E × E		Pb × Pb		Ps × Ps		E × Pb		(E × Pb) × (E × Pb)		Pb × Ps		Pb × (Pb × Ps)	
	*n*	$\hat{p}$	*n*	$\hat{p}$	*n*	$\hat{p}$	*n*	$\hat{p}$	*n*	$\hat{p}$	*n*	$\hat{p}$	*n*	$\hat{p}$
Failed to form pupae	12	0.07	20	0.07	0	0	2	0.07	7	0.03	0	0	2	0.02
Never emerged	18	0.1	4	0.01	4	0.13	3	0.1	17	0.08	1	0.03	1	0.01
Failed to emerge	14	0.08	9	0.03	3	0.1	1	0.03	8	0.04	0	0	1	0.01
Total failures	44	0.25	33	0.11	7	0.23	6	0.2	32	0.16	1	0.03	4	0.05
Emerged successfully	131	0.75	255	0.89	24	0.77	24	0.8	173	0.84	31	0.97	79	0.95
		(0.68, 0.81)		(0.84, 0.92)		(0.60, 0.89)		(0.63, 0.90)		(0.79, 0.89)		(0.84, 1.00)		(0.88, 0.98)
Total pupations	175		288		31		30		205		32		83	

### QUANTIFYING ISOLATION

We used Sobel and Chen's (2014) method of quantifying reproductive isolation to summarize under a single measure the strength of isolation caused by each of the barriers studied in our experiments. The results presented in Table 6 show strong sexual prezygotic isolation in the sympatric and parapatric pairs, whereas the allopatric pair shows weak sexual prezygotic isolation. Although the sympatric pair displays no postzygotic isolation, both allopatric and parapatric pairs show intermediate to high levels of postzygotic isolation mediated by female F1 sterility.

**Table 6:** Reproductive isolation, *Ri*, caused by different traits for each taxon pair. Pheromones, color pattern preference, and live courtship (underlined) each contribute to the overall “Mating” reproductive isolation. ^*^ Values of 1 assigned due to female hybrid sterility; ‐ no data available

	Prezygotic					
	Geography	Host plant	Pheromones	Color pattern preference	Live courtship	Mating
*H. elevatus* to *H. p. butleri*	−0.17	0.06	1	0.39	0.48	1
*H. elevatus* to *H. p. sergestus*	>0.99	0.24	1	0.21	0.5	1
*H. p. butleri* to *H. p. sergestus*	>0.99	−0.62	1	0.34	0.20	0.03

	Postzygotic					
	F1 hatch rate	F2 hatch rate	BC hatch rate	F1 pupal success	F2 pupal success	BC pupal success
*H. elevatus* to *H. p. butleri*	0.01	−0.02	−0.04	0.01	−0.01	–
*H. elevatus* to *H. p. sergestus*	–	1*	–	–	1*	–
*H. p. butleri* to *H. p. sergestus*	−0.02	1*	0.31	‐0.08	1*	0.27

^*^Values of 1 assigned due to female hybrid sterility.

## Discussion

Diverging populations in geographic contact typically exhibit prezygotic isolation and/or extrinsic postzygotic isolation, with no intrinsic postzygotic isolation (Coyne and Orr 2004, and see appendix of Chamberlain et al. (2009) for additional examples). Indeed, theory predicts that it is difficult for DMIs to evolve in the face of gene flow (Turelli et al. 2001; Bank et al. 2012), even leading some authors to claim that finding a lack of hybrid interfertility or inviability is a “litmus test” of sympatric speciation (Coyne and Orr 2004, p. 177). Here, we show that sympatric taxa (*H. elevatus* and *H. p. butleri*) show strong prezygotic isolation and that they rarely hybridize in captivity. Nonetheless, we also show, via forced matings, that the hybrids are completely fertile. In contrast, allopatric taxa separated by a narrow cordillera (*H. p. butleri* and *H. p. sergestus*) mate freely when brought together in captivity, even though female F1 hybrids are sterile. The parapatric taxa (*H. elevatus* and *H. p. sergestus*) exhibit both assortative mating and sterility of F1 female hybrids. These findings are summarized in Figure 2B. We now discuss each reproductive barrier in detail, before discussing the geography of divergence and species boundaries in these taxa.

### PREZYGOTIC ISOLATION

*Heliconius elevatus* and *H. p. butleri* exhibit fine scale habitat divergence and are only occasionally found together; populations of each are relatively scarce and patchy, inhabiting well‐drained forest versus seasonally flooded forest, respectively. Such habitat divergence is expected among sympatric species to prevent competitive exclusion (Hardin 1960), and consequently is also a requirement of models of sympatric speciation (van Doorn et al. 1998). *Heliconius p. sergestus*, meanwhile, inhabits the dry forests within its narrow endemic range, making it parapatric with *H. elevatus* and allopatric with *H. p. butleri* (although the two are usually separated by as little as 20 km near Tarapoto). Because we know *H. p. sergestus* is capable of crossing the cordillera separating it from *H. p. butleri*, it seems likely that its geographic isolation is maintained by divergent adaptations, rather than the simple barrier effect of the mountains alone (Sobel et al. 2010). Abiotic gradients may be one of the most common drivers of speciation across all the domains of life (Li et al. 2016), and aridity gradients in particular have been associated with divergence among other *Heliconius* species (Jiggins et al. 1996; Jiggins and Davies 1998; Arias et al. 2008).

*Heliconius elevatus* and *H. p. butleri* also exhibit divergent host plant use; while they use the same suite of *Passiflora*, they do so at different frequencies, with *H. elevatus* more specialized and favoring canopy vines. Divergence between *H. p. butleri* and *H. p. sergestus* seems much less, although still likely significant. Our experiments produced conflicting results regarding host plant use in *H. elevatus* and *H. p. sergestus*, with one experiment indicating them to be very different, and the other failing to find a difference. This contradictory result likely stems from different sets of host plants tested in each experiment (one being a small subset of the other). In particular, *H. elevatus*’ preferred host *P. *(Laurifoliae) sp. was not included in the second experiment. The result also hints that the genetic basis for host plant differences among these taxa may involve multiple loci or alleles. Because *Heliconius* inhabit and often mate in the vicinity of their host plants (Mallet 1984, 1986; Estrada and Gilbert 2010), host plant divergence between sympatric and parapatric divergence may contribute to speciation, as with other phytophagous insects (Bush 1969; Berlocher and Feder 2002). Furthermore, because these host plant and habitat‐based prezygotic barriers act earlier in the sequence of reproductive barriers, they may more strongly reduce gene flow than later‐acting barriers such as pheromones (Ramsey et al. 2003).

Males of *H. elevatus* show a strong preference for their own wing color pattern phenotype, confirming a role of color pattern in mate choice, as with other *Heliconius* species (Jiggins et al. 2001b; Chamberlain et al. 2009; Merrill et al. 2011a) and butterflies in general (Silberglied and Taylor 1973; Papke et al. 2007). This barrier appears unidirectional, because courting male *H. p. butleri* and *H. p. sergestus* do not discriminate between models of their own taxon and those of *H. elevatus* (Table 1; note that *H. p. butleri* does show a preference for its own phenotype over *H. p. sergestus*). Despite this, in controlled experiments neither *H. p. butleri* nor *H. p. sergestus* males ever mated with *H. elevatus* females (Table 3). Mating in butterflies is typically thought to involve long‐range visual searching by males, with females then responding to male pheromones at close range (Vane‐Wright and Boppré 1993). Female choice for male pheromones has been shown in *Heliconius* (Mérot et al. 2015; Darragh et al. 2017; Southcott and Kronforst 2018), and we found marked differences between the male sex pheromones of all three taxa (Fig. 5). However, the lack of matings among taxa may also be the result of males responding to species‐specific female sex pheromones, and we note that although *H. elevatus*, *H. p. butleri*, and *H. p. sergestus* males all approached live *H. elevatus* females at similar rates, *H. p. butleri* and *H. p. sergestus* males actively courted them less (Table S3, Fig. 6). Overall, our data suggest that prezygotic isolation is very strong among all three of our study taxa (Table 6), but the relative contributions of sexual or habitat‐related barriers depends on the geography of the taxa (Ramsey et al. 2003; Sobel et al. 2010; Sobel and Chen 2014).

### INTRINSIC POSTZYGOTIC ISOLATION

Despite strong prezygotic sexual isolation, sympatric *H. elevatus* and *H. p. butleri* have no detectable intrinsic postzygotic isolation (Table 6) and produce fertile hybrids. Prezygotic isolation without intrinsic incompatibilities is also found in several other closely related pairs of *Heliconius* species (McMillan et al. 1997; Kronforst et al. 2006; Chamberlain et al. 2009; Merrill et al. 2011a, 2015; Jiggins 2017).

In contrast, female hybrids from crosses between *H. p. sergestus* and either *H. elevatus* or *H. p. butleri* are sterile. In *Heliconius*, most previously documented cases of hybrid sterility are from crosses between the relatively divergent *H. melpomene* and *H. cydno* lineages, which show strong prezygotic isolation (Naisbit et al. 2002; Mérot et al. 2017). However, female hybrid sterility has also been documented among geographically distant populations of *H. melpomene* from Panama and French Guiana (Jiggins et al. 2001b). As the heterogametic sex in most Lepidoptera is the female (ZW), sex‐biased hybrid sterility is in accordance with Haldane's (1922) rule. This kind of intrinsic barrier is most readily explained via “dominance theory,” in which one or more epistatic partner loci in a DMI is recessive and found on the Z chromosome, and is thus exposed only in the heterogametic sex (Turelli and Orr 1995; Turelli and Moyle 2007). Z‐linked hybrid sterility has previously been confirmed in *Heliconius* (Jiggins et al. 2001a; Naisbit et al. 2002). We found no evidence for a reduction in hybrid viability, thus our results also conform to the general finding that hybrid sterility evolves before hybrid lethality (Presgraves 2002).

### REINFORCEMENT AND SPECIATION WITH GENE FLOW

A key prediction of reinforcement is that populations with the potential for gene flow should show higher sexual isolation than allopatric populations, and our data are broadly consistent with this (but see Noor 1999). We observed strong assortative mating between the sympatric *H. elevatus* and *H. p. butleri*, but not between the allopatric *H. p. sergestus* and *H. p. butleri*. Accordingly, the most divergent male sex pheromone profiles are also those of *H. p. butleri* and *H. elevatus*, with *H. p. sergestus* intermediate. In addition, *H. p. butleri* and *H. elevatus* males show a preference for courting females of their own taxon, whether presented with model wings or live females, whereas *H. p. sergestus* does not (Table 1, Fig. 6). Given the parapatric contact between *H. elevatus* and *H. p. sergestus* (with potential for intermediate levels of gene flow), we might expect that matings between *H. elevatus* and *H. p. sergestus* should be more common than matings Between *H. elevatus* and *H. p. butleri*. Unfortunately, the difficulty of achieving matings among the taxa mean we are unable to draw any conclusions in this respect.

If the strong reproductive isolation between *H. p. butleri* and *H. elevatus* in sympatry is indeed due to reinforcement, it is curious that the pair exhibits no apparent hybrid sterility. Instead, reinforcement is presumably driven by ecological postzygotic barriers and other ecological differences that cannot be measured using methods employed here. For example, in *Heliconius*, hybrids among taxa from different mimicry rings may suffer because they have intermediate, nonmimetic phenotypes (Figure 2B) that are vulnerable to predators (Merrill et al. 2012; Arias et al. 2016). A similar lack of correlation between sexual barriers and intrinsic postzygotic barriers is also found among sympatric species pairs of *Drosophila*, and is likely explained by unmeasured ecological factors (Turelli et al. 2014).

Formal estimates of gene flow between *H. elevatus* and *H. p. butleri* have yet to be calculated, but their interfertility and the existence of putative wild hybrids (Brower 2018, M. Joron, pers. comm.) suggest that at least part of the speciation process is taking place in the face of gene flow. However, while the pair are now unambiguously sympatric, it is unclear whether this has been the case throughout divergence (Losos and Glor 2003). Prezygotic isolation is presently very strong because multiple traits act in concert to reduce gene flow. If hybrid speciation was triggered by exchange of the rayed phenotype between *H. melpomene* and the ancestor of *H. elevatus* (*Heliconius* Genome Consortium 2012; Wallbank et al. 2016), rapid attainment of tight linkage disequilibrium among these traits would have been necessary to prevent erosion of mimicry and other species differences (Felsenstein 1981; Duenez‐Guzman et al. 2009; Butlin and Smadja 2018). One of the introgressed color pattern loci, *cortex*, is trapped in a fixed ∼400 kb inversion in *H. pardalinus*, with *H. elevatus* having apparently receiving its uninverted copy of the *cortex* color locus from a rayed form of *H. melpomene* (Jay et al. 2018). Reduced recombination between the inverted and uninverted chromosome could have aided rapid achievement of such tight linkage disequilibrium during putative hybrid speciation of *H. elevatus* (Noor et al. 2001; Feder et al. 2003). Moreover, tight linkage among color pattern, mating preference, and host plant use has been demonstrated in other *Heliconius* species (Kronforst et al. 2006; Merrill et al. 2011b, 2013, 2019). Nonetheless, it is perhaps more plausible that *H. elevatus* initially established itself in allopatry or parapatry (Duenez‐Guzman et al. 2009; Rosser et al. 2015). Conceivably, hybrid sterility might also have evolved between *H. elevatus* and *H. pardalinus* during this initial period, before being lost as a result of gene flow after secondary contact, leaving the peripherally distributed *H. p. sergestus* as an older relict of the ancestral *H. pardalinus*. This postspeciation introgression scenario would also explain the current genomic paraphyly of *H. pardalinus* relative to *H. elevatus* (Fig. 2A).

### SPECIES BOUNDARIES

Are *H. p. butleri*, *H. p. sergestus*, and *H. elevatus* two species? Or three? Or one? In the relaxed biological concept of many of today's ornithologists (Gill 2014), three species would almost certainly be recognized on the grounds that all the taxa display some sort of reproductive isolation from one another. We would agree that the sympatric *H. elevatus* and *H. p. butleri* are separate species because in nature, multiple prezygotic barriers allow them to maintain separate identities in sympatry across almost the entire Amazon drainage. Whether *H. p. sergestus* is a third distinct species is a more arbitrary decision. On the one hand, it seems likely that *H. p. sergestus* would merge with *H. p. butleri* if the two were to become sympatric, despite the sterility of hybrid females. On the other hand, their largely allopatric distributions appear to be maintained by adaptations to different habitats, and so they could be also be seen as reproductively isolated, and good species under the biological species concept (Sobel et al. 2010). For the moment, we follow the conservative species concept of most lepidopterists (and of *Heliconius* taxonomists, in particular [G. Lamas, in Jiggins 2017]) and continue to recognize *H. p. sergestus* and *H. p. butleri* as geographic subspecies within *H. pardalinus*. This accords with our treatment of other species of *Heliconius*, for example, *H. melpomene*, which also shows hybrid sterility among distant populations (Jiggins et al. 2001a). Although others may disagree with these standards, it is important to note that the current study is not biased by the particular species delimitation we have adopted here.

## Conclusions

In the 20^th^ century, both reinforcement and sympatric speciation were often considered unlikely for theoretical and empirical reasons (Mayr 1963; Felsenstein 1981; Barraclough and Vogler 2000). Concurrently, much speciation research focused on hybrid incompatibilities and sterility, perhaps because *Drosophila* offers such a tractable system with which to address such questions (Orr 2005). The present century has seen a change in attitudes toward speciation with gene flow (Bolnick and Fitzpatrick 2007), and a large body of research has developed focusing on prezygotic and extrinsic postzygotic isolation, and the role of ecology in speciation (Schluter 2009; Nosil 2012). Here, we present evidence suggesting important roles for both geographic isolation and gene flow during speciation, and furthermore our results highlight how the evolution of assortative mating and intrinsic postzygotic isolation may depend on geography.

Associate Editor: C. M. Smadja

Handling Editor: Mohamed A. F. Noor

## Supporting information


**Figure S1**. Experimental setup for the male colour pattern preference experiment.
**Figure S2**. Dotted lines illustrate the areas sampled for pheromone analysis for both the androconial and hind wing control regions in the three taxa.
**Figure S3**. Likelihood of courtship behaviors toward a) the *H. elevatus* color pattern by males of the two sympatric species, *H. elevatus* and *H. p. butleri*; b) the *H. elevatus* pattern by the two parapatric species*, H. elevatus* and *H. p. sergestus*; and c) the *H. p. butleri* pattern by the two allopatric sub‐species, *H. p. butleri* and *H. p. sergestus*.
**Figure S4**. Assay of courtship behaviors within and between taxa. Single virgin females were presented to groups of 15 males (5 of each taxon) and courtship behaviors (approach, hover or alighting) toward the females were recorded.
**Table S1**. Proportion of eggs laid on different host plants by *H. elevatus, H. p. butleri* and *H. p. sergestus* during the host plant experiment with 21 *Passiflora* species.
**Table S2**. Results from 1) the Wilcoxon signed rank test comparing the androconial region at the anterior margin of the hind wing and the non‐androconial region at the posterior margin of the hind wing. 2) Mann‐Whitney U test comparing the androconial region of males with the anterior margin of the hind wing in females. 3) Kruskal‐Wallis testing for differences in the amount of each compound in pairwise comparisons of taxa (not used for the determination of “putative pheromone” list).
**Table S3**. Statistical significance of pairwise comparisons between the numbers of courtship behaviors (Figures 6 and S4) made by males of each taxon towards females of a given taxon (top three columns, with the number of individual females tested in brackets).
**Table S4**. Pupation duration in days of the three taxa.
