# Supplementary material for: Geographic contrasts between pre‐ and postzygotic barriers are consistent with reinforcement in Heliconius butterflies
Source: Evolution. 2019 Sep 11;73(9):1821–38. doi: 10.1111/evo.13804 (PMC6771877; doi:10.1111/evo.13804)
Supplement: Supplementary file 1 — Figure S1. Experimental setup for the male colour pattern preference experiment. Figure S2. Dotted lines illustrate the areas sampled for pheromone analysis for both the androconial and hind wing control regions in the three taxa. Figure S3. Likelihood of courtship behaviors toward a) the H. elevatus color pattern by males of the two sympatric species, H. elevatus and H. p. butleri; b) the H. elevatus pattern by the two parapatric species, H. elevatus and H. p. sergestus; and c) the H. p. butleri pattern by the two allopatric sub‐species, H. p. butleri and H. p. sergestus. Figure S4. Assay of courtship behaviors within and between taxa. Single virgin females were presented to groups of 15 males (5 of each taxon) and courtship behaviors (approach, hover or alighting) toward the females were recorded. Table S1. Proportion of eggs laid on different host plants by H. elevatus, H. p. butleri and H. p. sergestus during the host plant experiment with 21 Passiflora species. Table S2. Results from 1) the Wilcoxon signed rank test comparing the androconial region at the anterior margin of the hind wing and the non‐androconial region at the posterior margin of the hind wing. 2) Mann‐Whitney U test comparing the androconial region of males with the anterior margin of the hind wing in females. 3) Kruskal‐Wallis testing for differences in the amount of each compound in pairwise comparisons of taxa (not used for the determination of “putative pheromone” list). Table S3. Statistical significance of pairwise comparisons between the numbers of courtship behaviors (Figures 6 and S4) made by males of each taxon towards females of a given taxon (top three columns, with the number of individual females tested in brackets). Table S4. Pupation duration in days of the three taxa. [file EVO-73-1821-s001.docx]

**SUPPLEMENTARY MATERIALS**


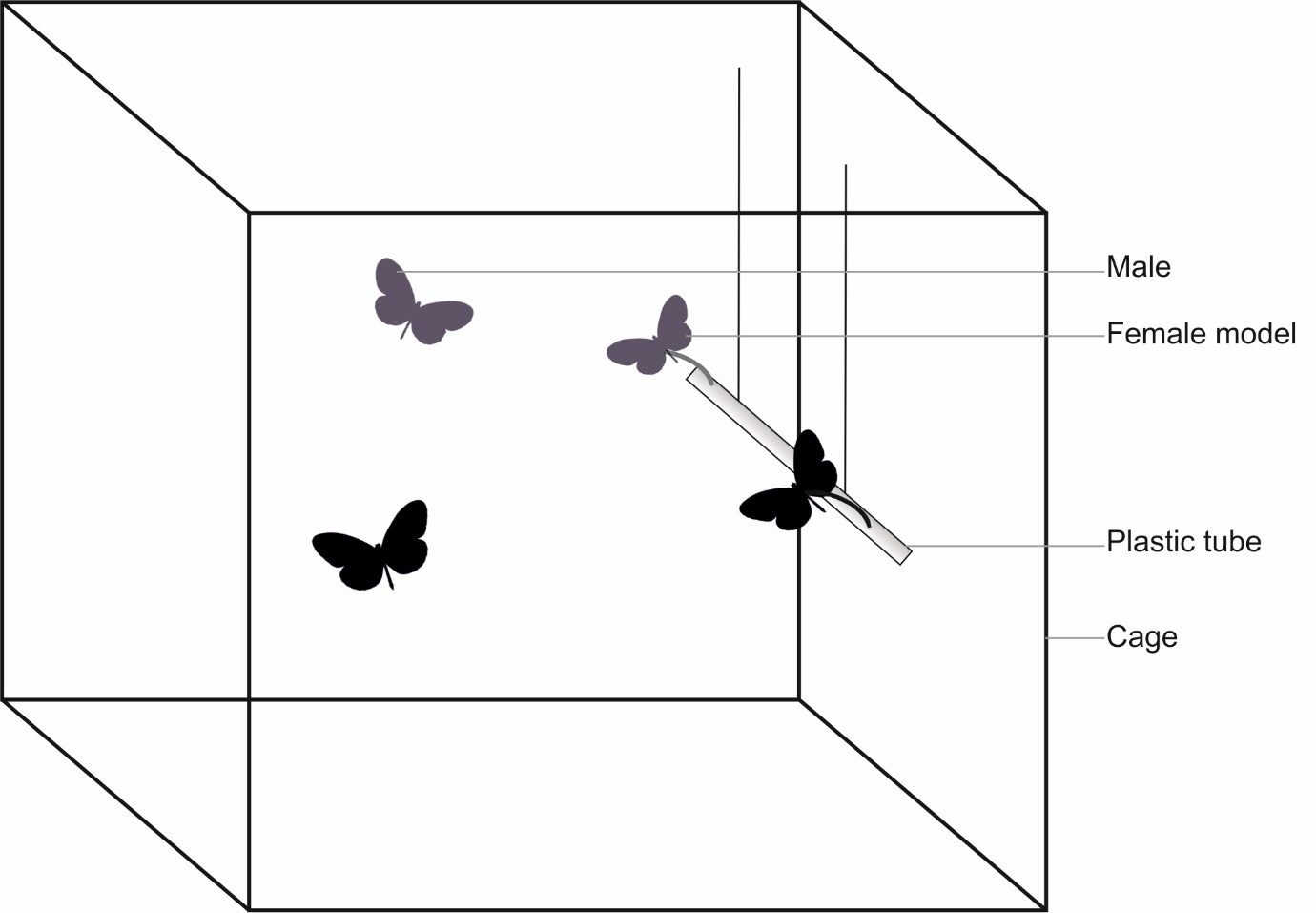


Figure S1. Experimental setup for the male colour pattern preference experiment. Males were left to fly freely in a cage approximately 2m (W) × 1.7m (L) × 1.7m (H). Female models were mounted on plastic cable ties and fixed to the ends of a 1m long plastic tube, hanging horizontally ~1.3m above the ground. Models comprised real female wings washed with dichloromethane to remove any potential pheromones and reinforced with paper. By pulling on a string tied to the tube in swift, short motions, an observer could cause the models to flutter in a flight-like motion, from outside of the cage.


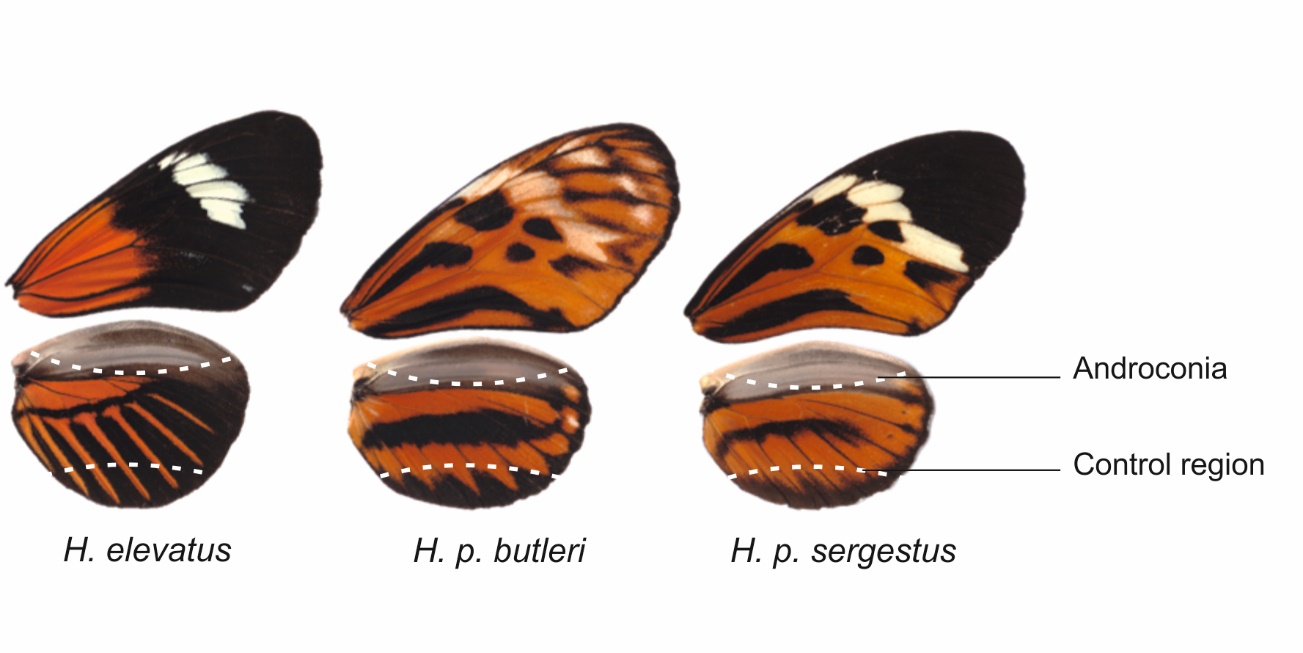


**Figure S2.** Dotted lines illustrate the areas sampled for pheromone analysis for both the androconial and hind wing control regions in the three taxa

**Figure S3.** Likelihood of courtship behaviours towards a) the *H. elevatus* colour pattern by males of the two sympatric species, *H. elevatus* and *H. p. butleri*; b) the *H. elevatus* pattern by the two parapatric species*, H. elevatus* and *H. p. sergestus*; and c) the *H. p. butleri* pattern by the two allopatric sub-species, *H. p. butleri* and *H. p. sergestus*. The horizontal dashed line represents no preference between the two patterns. Error bars correspond to the 95% confidence interval of the courtship likelihoods. Numbers of courtship behaviours observed by the different taxa is shown in bold, with the numbers of individuals performing the courtship behaviours in brackets.


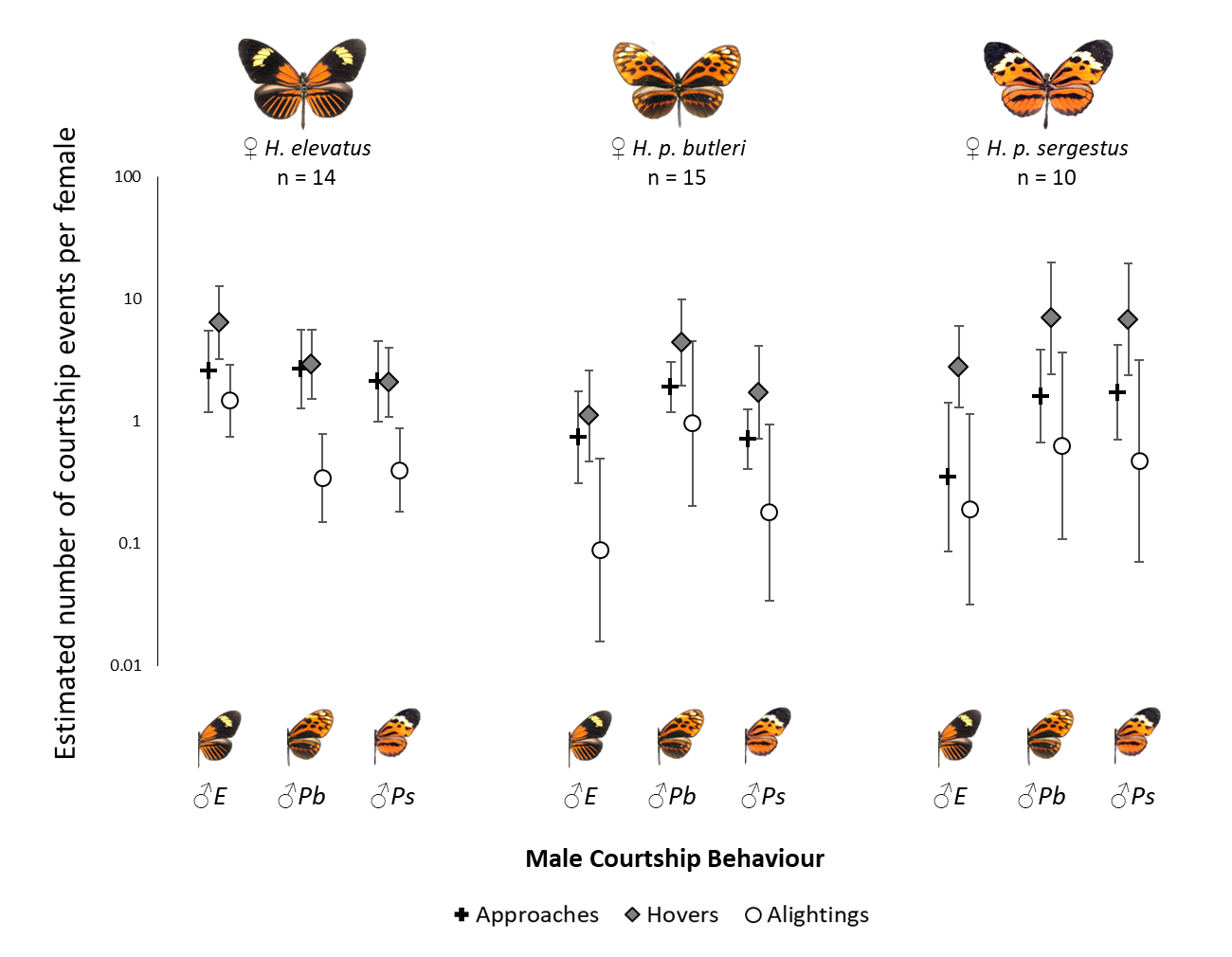


**Figure S4.** Assay of courtship behaviours within and between taxa. Single virgin females were presented to groups of 15 males (5 of each taxon) and courtship behaviours (approach, hover or alighting) toward the females were recorded. The y-axis gives the expected number of courtship behaviours per trial by males towards the female taxa, obtained from GLMMs (shown on a log_10_ scale). The statistical significance of the differences were also obtained from GLMM model outputs and are shown in Table S3. Error bars are 95% Wald confidence intervals. n is the number of virgin females tested of each taxon; *E* = *H. elevatus*, *Pb* = *H. p. butleri*, *Ps* = *H. p. sergestus*.

Table S1. Proportion of eggs laid on different host plants by *H. elevatus, H. p. butleri* and *H. p. sergestus* during the host plant experiment with 21 *Passiflora* species. Figures in brackets are the numbers of eggs laid by each taxon on each plant.

| **Host species** | ***H. elevatus*** | | ***H. p. butleri*** | | ***H. p. sergestus*** | |
| --- | --- | --- | --- | --- | --- | --- |
| *Passiflora (Plectostemma: Punctatae) sp.* | 0.02 | (3) | 0.02 | (7) | 0.02 | (3) |
| *Passiflora foetida* | 0.02 | (3) | 0.04 | (16) | 0.01 | (2) |
| *Passiflora laurifolia* | 0.05 | (9) | 0.06 | (27) | 0.2 | (31) |
| *Passiflora vitifolia* | 0.01 | (1) | 0.03 | (13) | 0 | (0) |
| *Passiflora trifasciata* | 0 | (0) | 0 | (0) | 0 | (0) |
| *Passiflora coriacea* | 0 | (0) | 0 | (0) | 0 | (0) |
| *Passiflora tarapotina* | 0.05 | (8) | 0.12 | (50) | 0.01 | (1) |
| *Passiflora hastifolia* | 0.08 | (14) | 0.01 | (6) | 0.01 | (1) |
| *P.* (Laurifoliae) sp. | 0.41 | (71) | 0.03 | (14) | 0 | (0) |
| *Passiflora ferruginea* | 0 | (0) | 0 | (0) | 0 | (0) |
| *Passiflora quadrangularis* | 0.01 | (1) | 0.03 | (14) | 0.06 | (9) |
| *Passiflora serratodigitata* | 0.14 | (24) | 0.14 | (58) | 0.24 | (36) |
| *Dilkea retusa* | 0 | (0) | 0 | (1) | 0 | (0) |
| *Passiflora spinosa* | 0.05 | (9) | 0.07 | (28) | 0 | (0) |
| *Passiflora menispermifolia* | 0 | (0) | 0 | (1) | 0 | (0) |
| *Passiflora edulis* | 0.11 | (19) | 0.24 | (102) | 0.38 | (58) |
| *Passiflora riparia* | 0.03 | (5) | 0.16 | (68) | 0.03 | (4) |
| *Passiflora triloba* | 0.01 | (1) | 0.01 | (6) | 0 | (0) |
| *Passiflora rubra* | 0 | (0) | 0 | (0) | 0 | (0) |
| *Passiflora auriculata* | 0 | (0) | 0 | (0) | 0 | (0) |
| *Passiflora skiantha* | 0.03 | (5) | 0.03 | (14) | 0.05 | (7) |
| **Total** | **1.00** | **(173)** | **1.00** | **(425)** | **1.00** | **(152)** |

**Table S2.** Results from 1) the Wilcoxon signed rank test comparing the androconial region at the anterior margin of the hindwing and the non-androconial region at the posterior margin of the hindwing. 2) Mann-Whitney U test comparing the androconial region of males with the anterior margin of the hindwing in females. 3) Kruskal-Wallis testing for differences in the amount of each compound in pairwise comparisons of taxa (not used for the determination of “putative pheromone” list). Significant results are presented in bold; compounds used in the final PCA are found in significantly higher concentrations in androconia of at least one taxon compared to controls. ? = unknown position of double bond.

|  |  |  |  |  |  |  |  |  |  | |  |  |  | |  |  | |  | |  | |  | |  |  | |  | | |
| --- | --- | --- | --- | --- | --- | --- | --- | --- | --- | --- | --- | --- | --- | --- | --- | --- | --- | --- | --- | --- | --- | --- | --- | --- | --- | --- | --- | --- | --- |
|  | **Compound** | **Total Amounts in Male Androconia, nmol** | | | | | | **Wilcoxon signed rank test** | | | | | | **Independent-Samples Mann-Whitney U Test** | | | | | **Kruskall Wallis** | | | | | | | **Compounds used for PCA analysis** | |  |  |
|  |  | ***H. p. butleri*** | | ***H. elevatus*** | | ***H. p. sergestus*** | | ***H. p. butleri*** | | ***H. elevatus*** | | ***H. p. sergestus*** | | ***H. p. butleri* - female control** | | | ***H. elevatus* - female control** | | ***H. p. butleri - H. elevatus*** | | ***H. p. butleri - H. p. sergestus*** | | ***H. elevatus - H. p. sergestus*** | | |  |  |  |  |
|  |  | **Median** | **Percentile 25,75** | **Median** | **Percentile 25,75** | **Median** | **Percentile 25,75** | **p value** | | **p value** | | **p value** | | **p value** | | | **p value** | | **p value** | | **p value** | | **p value** | | |  | |  |  |
|  | **Homovanillyl alcohol** | **0.091** | 0.067, 0.147 | **0.043** | 0.021, 0.093 | **0** | 0, 0.019 | **0.043** | | 0.109 | | 0.18 | | **0.038** | | | 0.121 | | 0.099 | | **0.047** | | 0.168 | | | **x** | |  |  |
|  | **Syringaaldehyde** | **0** | 0, 0 | **0** | 0, 0 | **0** | 0, 0.008 | 0.109 | | 0.317 | | 0.18 | | 0.069 | | | 0.758 | | 0.932 | | 0.532 | | 0.53 | | |  | |  |  |
|  | **Diterpene** | **0.026** | 0.019, 0.052 | **0.038** | 0.016, 0.075 | **0.025** | 0.023, 0.067 | 0.465 | | 0.225 | | 0.08 | | 0.069 | | | 0.273 | | 0.552 | | 0.84 | | 0.901 | | |  | |  |  |
|  | **Hexadecanal** | **0.005** | 0, 0.023 | **0** | 0, 0.003 | **0** | 0, 0 | 0.593 | | 0.317 | | 0.317 | | 0.305 | | | 1 | | **0.026** | | 0.416 | | 0.535 | | |  | |  |  |
|  | **Hexahydrofarnesylacetone** | **5.737** | 4.543, 6.906 | **0** | 0, 0 | **1.012** | 0.693, 1.053 | **0.043** | | 1 | | **0.043** | | **0.019** | | | 1 | | **0** | | **0.004** | | **0** | | | **x** | |  |  |
|  | **Nonadecane** | **0** | 0, 0 | **0** | 0, 0.011 | **0** | 0, 0 | 0.317 | | 0.273 | | 1 | | 1.000 | | | 0.606 | | 0.159 | | 0.535 | | 0.189 | | |  | |  |  |
|  | **?-Eicosene** | **0.034** | 0.02, 0.064 | **0** | 0, 0 | **0** | 0, 0 | **0.043** | | 1 | | 0.317 | | **0.019** | | | 1 | | **0** | | **0.003** | | 0.157 | | | **x** | |  |  |
|  | **Eicosane** | **0.003** | 0, 0.008 | **0.042** | 0.009, 0.105 | **0** | 0, 0 | 0.18 | | 0.068 | | 0.317 | | 0.571 | | | 0.121 | | **0.015** | | 0.615 | | **0.022** | | |  | |  |  |
|  | **??-Heneicosadiene** | **0.028** | 0.013, 0.036 | **0** | 0, 0.014 | **0.001** | 0, 0.007 | **0.043** | | 0.317 | | 0.157 | | **0.038** | | | 0.606 | | **0.012** | | **0.011** | | 0.942 | | | **x** | |  |  |
|  | **(*Z*)-9-Heneicosene** | **8.697** | 7.522, 12.743 | **0.005** | 0, 0.042 | **1.249** | 1.178, 2.431 | **0.043** | | 1 | | **0.043** | | **0.019** | | | 0.364 | | **0** | | **0.001** | | **0.002** | | | **x** | |  |  |
|  | **1-Octadecanol** | **0** | 0, 0.001 | **0.009** | 0, 0.019 | **0** | 0, 0 | 0.317 | | 0.18 | | 1 | | 0.8 | | | 0.273 | | 0.093 | | 0.367 | | **0.037** | | |  | |  |  |
|  | **1-Heneicosene** | **0** | 0, 0.046 | **0** | 0, 0 | **0** | 0, 0 | 1 | | 0.317 | | 1 | | 0.571 | | | 0.485 | | 0.183 | | 0.176 | | 0.48 | | |  | |  |  |
|  | **Heneicosane** | **0.084** | 0.05, 0.123 | **12.994** | 8.08, 15.511 | **0.088** | 0.062, 0.121 | **0.043** | | **0.043** | | **0.043** | | **0,038** | | | **0.030** | | **0** | | 0.882 | | **0.002** | | | **x** | |  |  |
|  | **?-Docosene** | **0.104** | 0.062, 0.155 | **0** | 0, 0 | **0.031** | 0.028, 0.042 | **0.043** | | 1 | | 0.068 | | **0,019** | | | 1 | | **0** | | **0.007** | | **0.002** | | | **x** | |  |  |
|  | **Oleyl acetate** | **0.082** | 0.059, 0.096 | **0** | 0, 0 | **0** | 0, 0.008 | 0.068 | | 1 | | 0.18 | | **0,038** | | | 1 | | **0** | | **0.004** | | **0.038** | | | **x** | |  |  |
|  | **(*Z*)-11-Eicosenal** | **0.018** | 0, 0.058 | **0** | 0, 0 | **0.051** | 0.048, 0.052 | 0.109 | | 0.317 | | 0.068 | | 0,229 | | | 0.485 | | **0.037** | | 0.449 | | **0.023** | | |  | |  |  |
|  | **Docosane** | **0.009** | 0, 0.016 | **0.117** | 0.074, 0.205 | **0** | 0, 0 | 0.285 | | 0.08 | | 0.317 | | 0,171 | | | 0.061 | | **0.001** | | **0.016** | | **0.004** | | |  | |  |  |
|  | **Octadecyl acetate** | **0.053** | 0.035, 0.059 | **0** | 0, 0 | **0.012** | 0, 0.018 | 0.068 | | 1 | | 0.285 | | **0,038** | | | 0.364 | | **0** | | **0.025** | | **0.009** | | | **x** | |  |  |
|  | **Phytol** | **0.089** | 0.023, 0.13 | **0** | 0, 0 | **0.048** | 0.029, 0.087 | **0.043** | | 0.317 | | 0.068 | | **0,038** | | | 1 | | **0** | | 0.459 | | **0.004** | | | **x** | |  |  |
|  | **Eicosanal** | **0** | 0, 0 | **0** | 0, 0 | **0** | 0, 0 | 0.18 | | 1 | | 1 | | 0.687 | | | 0.485 | | 0.348 | | 0.255 | | 0.48 | | |  | |  |  |
|  | **(*Z*)-11-Eicosenol** | **0.619** | 0.43, 0.76 | **0.048** | 0, 0.075 | **0** | 0, 0 | 0.109 | | 0.317 | | 1 | | 0.076 | | | 0.273 | | **0.002** | | **0.005** | | **0.038** | | |  | |  |  |
|  | **(*Z*)-9-Tricosene** | **0.832** | 0.442, 1.172 | **0.024** | 0.017, 0.059 | **0.262** | 0.218, 0.303 | **0.043** | | 0.225 | | **0.043** | | **0.019** | | | **0.030** | | **0** | | **0.02** | | **0.002** | | | **x** | |  |  |
|  | **Tricosane** | **0.031** | 0.026, 0.059 | **0.914** | 0.577, 1.243 | **0.048** | 0.04, 0.054 | **0.043** | | **0.043** | | **0.043** | | 0.114 | | | **0.030** | | **0** | | 0.285 | | **0.002** | | | **x** | |  |  |
|  | **11-Methyltricosane** | **0** | 0, 0.02 | **0.006** | 0, 0.016 | **0.018** | 0.012, 0.022 | 0.18 | | 0.465 | | 0.068 | | 0.476 | | | 0.758 | | 0.81 | | 0.367 | | 0.606 | | |  | |  |  |
|  | **(*Z*)-11-Eicosenyl acetate** | **20.632** | 17.03, 22.131 | **0** | 0, 0 | **2.447** | 1.596, 2.465 | **0.043** | | 1 | | **0.043** | | **0.019** | | | 1 | | **0** | | **0.001** | | **0.001** | | | **x** | |  |  |
|  | **Tetracosane** | **0.027** | 0, 0.054 | **0.102** | 0.073, 0.148 | **0** | 0, 0 | 0.18 | | **0.043** | | 0.317 | | 0.229 | | | 0.606 | | **0.003** | | **0.03** | | **0.002** | | | **x** | |  |  |
|  | **Eicosyl acetate** | **0.123** | 0.07, 0.183 | **0** | 0, 0 | **0** | 0, 0 | 0.068 | | 1 | | 1 | | 0.076 | | | 1 | | **0** | | **0.005** | | 1 | | |  | |  |  |
|  | **(*Z*)-11-Eicosenyl propionate** | **2.08** | 1.6, 2.703 | **0** | 0, 0 | **0** | 0, 0 | **0.043** | | 1 | | 1 | | **0.019** | | | 1 | | **0** | | **0.001** | | 1 | | | **x** | |  |  |
|  | **?-Heneicosenyl_acetate** | **0** | 0, 0.061 | **0** | 0, 0 | **0** | 0, 0 | 0.18 | | 1 | | 1 | | 0.381 | | | 1 | | **0.016** | | 0.078 | | 1 | | |  | |  |  |
|  | **Pentacosane** | **0.109** | 0.053, 0.134 | **0.346** | 0.266, 0.419 | **0.158** | 0.111, 0.165 | **0.043** | | **0.043** | | 0.138 | | 0.229 | | | 0.485 | | **0.001** | | 0.299 | | **0.01** | | | **x** | |  |  |
|  | **11-Methylpentacosane** | **0.119** | 0.076, 0.208 | **0.638** | 0.258, 0.703 | **0.273** | 0.137, 0.312 | 0.08 | | **0.043** | | 0.345 | | 0.229 | | | 0.758 | | **0.005** | | 0.152 | | 0.22 | | | **x** | |  |  |
|  | **(*Z*)-13-Docosenyl acetate** | **0.043** | 0.017, 0.076 | **0** | 0, 0 | **0** | 0, 0 | 0.068 | | 1 | | 1 | | **0.038** | | | 1 | | **0** | | **0.003** | | 1 | | | **x** | |  |  |
|  | **Hexacosane** | **0.036** | 0.025, 0.066 | **0.123** | 0.08, 0.144 | **0.073** | 0.049, 0.074 | 0.138 | | **0.043** | | 0.068 | | 0.476 | | | 1 | | **0.004** | | 0.655 | | **0.032** | | | **x** | |  |  |
|  | **11-Methylhexacosane** | **0** | 0, 0.004 | **0.026** | 0.004, 0.044 | **0.033** | 0.029, 0.036 | 0.109 | | **0.043** | | 0.144 | | 0.686 | | | 1 | | **0.007** | | **0.006** | | 0.755 | | | **x** | |  |  |
|  | **Heptacosane** | **0.164** | 0.119, 0.236 | **0.737** | 0.625, 0.829 | **0.375** | 0.368, 0.501 | 0.225 | | **0.043** | | 0.08 | | 0.114 | | | 0.182 | | **0** | | **0.01** | | **0.01** | | | **x** | |  |  |
|  | **11-Methylheptacosane** | **0.049** | 0.039, 0.064 | **0.114** | 0.055, 0.143 | **0.058** | 0.054, 0.064 | **0.043** | | 0.225 | | 0.465 | | **0.019** | | | 1 | | 0.057 | | 0.687 | | 0.11 | | | **x** | |  |  |
|  | **Octacosane** | **0.014** | 0.006, 0.079 | **0.064** | 0.042, 0.109 | **0.046** | 0.021, 0.051 | 0.465 | | **0.043** | | 0.465 | | 0.571 | | | 0.909 | | 0.126 | | 0.96 | | 0.094 | | | **x** | |  |  |
|  | **Hexacosanal** | **0** | 0, 0 | **0.01** | 0, 0.059 | **0.043** | 0, 0.071 | 1 | | 0.068 | | 0.109 | | 1 | | | 0.909 | | **0.045** | | **0.036** | | 0.796 | | |  | |  |  |
|  | **Nonacosane** | **0.25** | 0.178, 0.285 | **0.519** | 0.458, 0.606 | **0.251** | 0.235, 0.347 | 0.08 | | **0.043** | | 0.5 | | 0.229 | | | 0.485 | | **0** | | 0.587 | | **0.01** | | | **x** | |  |  |
|  | **Octacosanal** | **0.006** | 0, 0.066 | **0.207** | 0.083, 0.602 | **0.331** | 0.141, 0.382 | **0.043** | | 0.08 | | 0.345 | | 0.800 | | | 0.758 | | **0.004** | | **0.047** | | 0.903 | | | **x** | |  |  |
|  |  |  |  |  |  |  |  |  |  | |  |  |  | |  |  | |  | |  | |  | |  |  | |  | | |

**Table S3.** Statistical significance of pairwise comparisons between the numbers of courtship behaviours (Figures 6 and S4) made by males of each taxon towards females of a given taxon (top three columns, with the number of individual females tested in brackets). E = *H. elevatus* males, Pb = *H. p. butleri* males, Ps = *H. p sergestus* males, with n = the total number of courtships observed (horizontally) and expected number of courtships determined from the GLMM in brackets (vertically) from each male taxa. *p*-values < 0.05 are in bold, and those no longer significant after a Bonferroni correction for 27 tests (*p* < 0.0019) are marked with a †.

|  | **♀ *H. elevatus (14)*** | | | | | ***♀ H. p butleri (15)*** | | | | | ***♀ H. p. sergestus (10)*** | | | | |
| --- | --- | --- | --- | --- | --- | --- | --- | --- | --- | --- | --- | --- | --- | --- | --- |
|  |  |  | **♂E** | **♂Pb** | **♂Ps** |  |  | **♂E** | **♂Pb** | **♂Ps** |  |  | **♂E** | **♂Pb** | **♂Ps** |
| **Approach** |  |  | n = 52 | n = 87 | 2.13 |  |  | n = 25 | n = 64 | n = 24 |  |  | n = 10 | n = 41 | n = 39 |
|  | **♂E** | (2.58) |  | 0.923 | 0.616 | **♂E** | (0.74) |  | **0.0001** | 0.907 | **♂E** | (0.35) |  | **0.001** | **0.0004** |
|  | **♂Pb** | (2.68) |  |  | 0.546 | **♂Pb** | (1.92) |  |  | **0.001** | **♂Pb** | (1.61) |  |  | 0.831 |
|  | **♂Ps** | (2.13) |  |  |  | **♂Ps** | (0.72) |  |  |  | **♂Ps** | (1.73) |  |  |  |
|  |  |  | **♂E** | **♂Pb** | **♂Ps** |  |  | **♂E** | **♂Pb** | **♂Ps** |  |  | **♂E** | **♂Pb** | **♂Ps** |
| **Hover** |  |  | n = 150 | n = 66 | n = 45 |  |  | n = 27 | n = 112 | n = 50 |  |  | n = 28 | n = 70 | n = 68 |
|  | **♂E** | (6.45) |  | **0.018^†^** | **0.001** | **♂E** | (1.11) |  | **0.001** | 0.32 | **♂E** | (2.8) |  | 0.088 | 0.098 |
|  | **♂Pb** | (2.93) |  |  | 0.337 | **♂Pb** | (4.4) |  |  | **0.02** | **♂Pb** | (7) |  |  | 0.955 |
|  | **♂Ps** | (2.1) |  |  |  | **♂Ps** | (1.73) |  |  |  | **♂Ps** | (6.8) |  |  |  |
|  |  |  | **♂E** | **♂Pb** | **♂Ps** |  |  | **♂E** | **♂Pb** | **♂Ps** |  |  | **♂E** | **♂Pb** | **♂Ps** |
| **Alighting** |  |  | n = 30 | n = 7 | n = 8 |  |  | n = 3 | n = 29 | n = 8 |  |  | n = 3 | n = 11 | n = 6 |
|  | **♂E** | (1.48) |  | **0.001** | **0.001** | **♂E** | (0.09) |  | **0.003^†^** | 0.445 | **♂E** | (0.19) |  | 0.181 | 0.352 |
|  | **♂Pb** | (0.35) |  |  | 0.787 | **♂Pb** | (0.97) |  |  | **0.014^†^** | **♂Pb** | (0.63) |  |  | 0.728 |
|  | **♂Ps** | (0.4) |  |  |  | **♂Ps** | (0.18) |  |  |  | **♂Ps** | (0.47) |  |  |  |

**Table S4.** Pupation duration in days of the three taxa.
n = number of pupae measured; p̂ = proportion of total pupae eclosing after X days.

| ***H. elevatus*** | | | ***H. p. butleri*** | | | ***H. p. sergestus*** | | |
| --- | --- | --- | --- | --- | --- | --- | --- | --- |
| **Duration** | **n** | **p̂** | **Duration** | **n** | **p̂** | **Duration** | **n** | **p̂** |
| 6 days | 0 | 0.00 | 6 days | 3 | 0.01 | 6 days | 0 | 0.00 |
| 7 days | 2 | 0.01 | 7 days | 38 | 0.11 | 7 days | 0 | 0.00 |
| 8 days | 88 | 0.58 | 8 days | 239 | 0.66 | 8 days | 30 | 0.57 |
| 9 days | 56 | 0.37 | 9 days | 75 | 0.21 | 9 days | 21 | 0.40 |
| 10 days | 4 | 0.03 | 10 days | 4 | 0.01 | 10 days | 2 | 0.04 |
| 11 days | 1 | 0.01 | 11 days | 2 | 0.01 | 11 days | 0 | 0.00 |
| **Total** | **151** |  | **Total** | **361** |  | **Total** | **53** |  |
| **Median days** | **8** |  | **Median days** | **8** |  | **Median days** | **8** |  |

## Supplementary Information S1. Phylogenetic reconstruction

Restriction site associated DNA (RAD) sequences for the Peruvian silvaniform taxa (*H. p. butleri* (n = 6), *H. p.* *sergestus* (n =5), *H. elevatus* (n =5), *H. ethilla* (n =3), *H. hecale* (n =5) and *H. numata* (n =6)) published by the (*Heliconius* Genome Consortium 2012) were used for maximum-likelihood (ML) phylogenetic inference, with an *H. melpomene aglaope* specimen included as the outgroup. Fastq files were downloaded from the ENA (ERP000991) and aligned to the closest available reference genome; *H. melpomene* Hmel2.5 (Davey et al. 2016), using BWA mem (Li and Durbin 2009). Bam files were sorted with Samtools (Li et al. 2009), and PCR duplicates marked with Picard-tools v1.100 MarkDuplicates (broadinstitute.github.io/picard). HaplotypeCaller from the GATK v3.8.0 (McKenna et al. 2010) was used in ERC mode with default settings to produce gVCF files. Genotypes were inferred using GATK GenotypeGVCFs, and the resulting vcf file processed using GATK VariantsToTable and quality filtered using a custom perl script. Indels were removed and low quality genotypes (genotypes with > 100× coverage, GQ < 30 and SNPQual < 30, MQ < 20) were marked as missing in the final genotype calls file. The final 6844298bp alignment for 31 samples was input into RAxML (Stamatakis 2014), and ML phylogenetic inference with 100 bootstrap replicates conducted using the GTRCAT model.

**Supplementary Information S2. Hand pairing.**

In order to achieve matings between particular butterflies, or to obtain interspecific matings when behavioural isolation is strong, some *Heliconius* may be forcibly mated using a technique called hand pairing (Clarke and Sheppard 1956). This approach involves holding a female and male in either hand and pressing their genitalia together, and is thought to work well for *H. numata*, but not for other *Heliconius* species (Sheppard 1963; Brown and Benson 1974). We found that *H. elevatus* could be paired to *H. pardalinus*, but with a low success rate. Nonetheless, the strategy was critical to the present study, as obtaining even within-species matings was difficult. The best approach seems to try for a short time with a number of different males, rather than repeatedly attempting with a single male, as this is rarely successful and may result in damage to the butterfly. The success rate seemed higher when using wild males and close to dusk, but matings were achieved at any time of day and under a broad range of weather conditions.

**Supplementary Information S6. Methods for calculating strength of reproductive isolation**

We followed the method presented by Sobel and Chen (2014) to quantify the level of reproductive isolation (*Ri*) caused by each trait, using the formula:

$$Ri=1-2x$$

The probability of gene flow *x* can be calculated using the numbers of heterospecific (H) and conspecific (C) matings in the case of prezygotic barriers, or with values of hybrid or pure species survival as H and C, respectively, for postzygotic barriers. In the case of barriers relating to encounter rates, these values can be replaced with measures of “unshared” (U) or “shared” (S) habitat, for example:

$$x=\frac{H}{H+C}=\frac{S}{U+S}$$

The strength of reproductive isolation caused can therefore be calculated and compared between different traits.

***Pre-zygotic isolation: habitat isolation***: To measure the probability of gene flow from co-occurrence across the taxon’s geographic range, we calculated the area of the polygons extrapolated from collection records (from Rosser et al. (2012, 2015)) to determine shared and unshared distribution.

***Pre-zygotic isolation: female host plant preference:*** Pianka’s similarity index from the first host plant experiment was used as a measure of *x*. *Ri* was not calculated from the second host plant experiment, as there were too few plant species were tested to accurately measure overlap.

***Pre-zygotic isolation: male colour pattern preference:*** To measure *x*, we subtracted the calculated probabilities of conspecific courtship from 1 (see values in Table 1) to get the probabilities of heterospecific courtship, and averaged the values for the two taxa used in each pairwise comparison.

***Pre-zygotic isolation: male sex pheromones:*** We used the area of overlap between the clusters produced by NMDS ordination to estimate *x*. As there was no overlap between the NMDS clusters this, resulted in values of *Ri* of 1.

***Pre-zygotic isolation: assortative mating:*** We calculated *x* using the total numbers of conspecific and heterospecific matings for pairs of taxa (Table 3). An *Ri* of 1 was calculated for *H. elevatus* as there were no heterospecific matings between *H. elevatus* and either of the other taxa.

***Pre-zygotic isolation: male courtship behaviours:*** We used hovers to calculate the *Ri* of courtship with live females, as this is considered the most reliable measure. We combined the total number of conspecific and heterospecific hovers from males performed on the females of the respective taxa to calculate *x*.

***Post-zygotic isolation: egg hatch rate and pupal survivorship:*** To establish pure species success rates for hatching and pupal survival, we averaged the rates of the pure crosses. For “between taxon” crosses, we averaged the success rates of every cross direction available. When crosses involved sterile females (success rate = 0), the values of Ri were 1. In backcrosses to pure species with either fertile males F1s or sterile female F1s, we first averaged the rates of all fertile crosses and then averaged this rate with the rate of crosses between the sterile females to pure species (success rate = 0).

**Supplementary Information S4. Notes on the host plant use of *Heliconius elevatus* and *Heliconius pardalinus***

In northern Peru, we observed wild *H. elevatus* investigating and ovipositing on *P. laurifolia* near the district of Shapaja on numerous occasions (in 1986, and 2012-2016). The only previously published host plant record for *H. elevatus* was also *P. laurifolia* (Colombia; Benson et al. (1975)). *Heliconius elevatus* larvae were also found on *P. coccinea* (km 47.5 Tarapoto-Yurimaguas, 2011), and *P. vitifolia* (Uruhuasha, 2013, pers. comm. by M. Chouteau), and the species has been seen investigating *Passiflora* sp. aff. *spinosa* (near Shapaja, 2016). However, the most important plant for *H. elevatus* in the Cordillera Escalera appears to be *Passiflora* (Laurifoliae) sp., a large-leaved canopy growing species with characteristic purple stems, which we presume to be undescribed, and whose presence seems to predict that of *H. elevatus*. We have observed *H. elevatus* investigating this plant at km 47.5 Tarapoto-Yurimaguas, and reared *H. elevatus* from a larva collected on it at El Tunel. In northern Brazil (Roraima), Suriname and French Guiana, *H. elevatus* and its close relative *H. luciana* also use a canopy vine; *P. longiracemosa*. We have also observed apparent associations between *H. elevatus* and *P. coccinea* in Brazil (Amazonas – Careiro Castanho) and southern Peru (Puerto Maldonado).

We have noticed a very strong association between *H. p. sergestus* and *Passiflora laurifolia* in the Mayo and Huallaga valleys (observations made at Ricuricocha, near Tarapoto, near Bella Vista, near Buenos Aires). There are multiple morphs of *Passiflora laurifolia* in the region, and those found at dry forest sites such as Ricuricocha have very distinct floral and fruit morphologies from those at wetter sites near Tarapoto. It is unclear whether they should be recognised as different species. We have reared *H. p. sergestus* from eggs or larvae found on these plants both at Ricuricocha and near Tarapoto. They are likely the primary host plants of *H. p. sergestus* across much of its range, especially in dry forests, as *Passiflora* species richness at these sites (where *H. p. sergestus* is also most common) is low, and there are no other candidate host species. In the Amazon lowlands, at Shuchushyacu, *H. p. butleri* is known to use yet another variant of *P. laurifolia*, and probably uses *P. coccinea* at Sangamayoc. *Heliconius pardalinus* (race unknown) was recorded using *P. spinosa* in San Martin (JM, 1984). Near Pucallpa, the local race (*H. p. dilatus*) likely uses a *P. coccinea*-like species and / or *P.* aff. *spinosa*. Previous published records suggest that lowland races of *H. pardalinus* from Brazil use *P. coccinea*, *P. nitida* and *P. spinosa* (Benson et al. 1975).

**Supplementary Information S5. Additional details of pheromone compounds.**

Most compounds detected in the three taxa are fatty acid derivatives ranging in chain length from C20 to C29, with a few additional, likely plant derived compounds (table S4). The two species have very different pheromone blend components: *H. elevatus* has a relatively simple blend comprising highly concentrated long-chained alkanes starting from icosane (20C), accompanied by methylated alkanes starting from 11-methyltricosane (24C) and very low concentrations of alkenes (20-23C). Of these alkanes, henicosane (21C) is the most prominent, with a mean concentration of 11.83 nmol/μl across the 10 male androconia. In comparison tricosane, the second most prominent alkane, has a mean concentration of 1.03 nmol/μl. In contrast, *H. p.* *butleri* has a complex blend that includes high concentrations of alkenes (C20-23), esters, phytol derivatives, the aromatic compounds homovanillyl alcohol and syringaldehyde, alcohols and aldehydes, with alkanes being found in very low concentrations. The *H. p. sergestus*blend is similar to that of *H. p.* *butleri* in terms of the most abundant compounds (hexahydrofarnesyl acetone, the ester (*Z*)-11-icosenyl acetate and the two alkenes (*Z*)-9-henicosene and (*Z*)-9-tricosene), but the latter compound was at lower concentrations than in *H. p. butleri.* The *H. p. sergestus* blend also contains fewer classes of molecules than *H. p. butleri*. While the most prominent *H. p. butleri* compounds are all found in *H. p. sergestus,* the latter lacks C_20_ and C_22_ alkenes, some esters, including (*Z*)-11-icosenyl propionate and (*Z*)-13-docosenyl acetate, and alcohols such as (*Z*)-11-icosenol which were nearly always detected in *H. p butleri*.  Alkanes typical of *H. elevatus* such as henicosane and tricosane are present in *H. p. sergestus*, although at much lower titres. Variable loadings of the nine compounds that explained the most variation are presented in S6b.

**Supplementary Information S6. Notes on the immature stages and reproductive biology of *Heliconius elevatus* and *Heliconius pardalinus***

When mated with males of their own taxon, *H. elevatus* and *H. p. sergestus* females have similar fecundities, laying on average 1.9 (0.8-3.0, n = 4) and 1.85 (0.2-3.5, n = 3) eggs per day, respectively (95% confidence intervals and number of butterflies tested in brackets). However, *H. p. butleri* females had higher fecundity, laying 3.9 (3.1-4.7, n = 10) eggs per day. Correspondingly, *H. elevatus-H. p. butleri* F1 females had intermediate fecundity of 2.8 (1.1-4.5) eggs per day; estimated using females mated either by F1 (n=4) or *H. elevatus* (n=1) males. *Heliconius p. sergestus* eggs took 3.0 days (2.6-3.4, n=26) to hatch, *H. p. butleri* eggs took 3.9 days (3.8-3.9, n=525), and *H. elevatus* took 4.0 days (3.9-4.2, n=118). The mean number of days from hatching to pupation was 18.2 days (17.6-18.8, n=41) for *H. p. butleri*, and 18.3 days (15.5-21.2, n=3) for *H. elevatus* (no data available for *H. p. sergestus*)*.* Final instar larvae of *H elevatus* and *H. pardalinus* are near identical, but *H. elevatus* has a more orange-red head capsule than *H. pardalinus*, which is more orange-yellow. The mean pupation time did not differ between taxa and was 8.12 days (8.05-8.19, n=361) for *H. p. butleri*, 8.47 days (8.31-8.63, n=53) for *H. p. sergestus* and 8.43 days (8.33-8.53, n=151) for *H. elevatus* (see also Table S4).

**References**

Benson, W. W., K. S. Brown, and L. E. Gilbert. 1975. Coevolution of plants and herbivores: passion flower butterflies. Evolution 29:659–680.

Brown, K. S., and W. W. Benson. 1974. Adaptive Polymorphism Associated with Multiple Müllerian Mimicry in Heliconius numata (Lepid. Nymph.). Biotropica 6:205–228.

Clarke, C. A., and P. M. Sheppard. 1956. Handpairing of butterflies. Lepidoptera News 10:47–53.

Davey, J. W., M. Chouteau, S. L. Barker, L. Maroja, S. W. Baxter, F. Simpson, R. M. Merrill, M. Joron, J. Mallet, K. K. Dasmahapatra, and C. D. Jiggins. 2016. Major Improvements to the Heliconius melpomene Genome Assembly Used to Confirm 10 Chromosome Fusion Events in 6 Million Years of Butterfly Evolution. G3 Genes Genomes Genetics 6:695–708.

*Heliconius* Genome Consortium. 2012. Butterfly genome reveals promiscuous exchange of mimicry adaptations among species. Nature 487:94–98.

Li, H., and R. Durbin. 2009. Fast and accurate short read alignment with Burrows–Wheeler transform. Bioinformatics 25:1754–1760.

Li, H., B. Handsaker, A. Wysoker, T. Fennell, J. Ruan, N. Homer, G. Marth, G. Abecasis, R. Durbin, and 1000 Genome Project Data Processing Subgroup. 2009. The Sequence Alignment/Map format and SAMtools. Bioinformatics 25:2078–2079.

McKenna, A., M. Hanna, E. Banks, A. Sivachenko, K. Cibulskis, A. Kernytsky, K. Garimella, D. Altshuler, S. Gabriel, M. Daly, and M. A. DePristo. 2010. The Genome Analysis Toolkit: A MapReduce framework for analyzing next-generation DNA sequencing data. Genome Res. 20:1297–1303.

Rosser, N., K. M. Kozak, A. B. Phillimore, and J. Mallet. 2015. Extensive range overlap between heliconiine sister species: evidence for sympatric speciation in butterflies? BMC Evol. Biol. 15:125.

Rosser, N., A. B. Phillimore, B. Huertas, K. R. Willmott, and J. Mallet. 2012. Testing historical explanations for gradients in species richness in heliconiine butterflies of tropical America. Biol. J. Linn. Soc. 105:479–497.

Sheppard, P. M. 1963. Some genetic studies of Müllerian mimics in butterflies of the genus *Heliconius*. Zool. NY 48:145–154.

Sobel, J. M., and G. F. Chen. 2014. Unification of methods for estimating the strength of reproductive isolation. Evolution 68:1511–1522.

Stamatakis, A. 2014. RAxML version 8: a tool for phylogenetic analysis and post-analysis of large phylogenies. Bioinformatics 30:1312–1313.
